# A Modular Plasmid Assembly Kit for Multigene Expression, Gene Silencing and Silencing Rescue in Plants

**DOI:** 10.1371/journal.pone.0088218

**Published:** 2014-02-13

**Authors:** Andreas Binder, Jayne Lambert, Robert Morbitzer, Claudia Popp, Thomas Ott, Thomas Lahaye, Martin Parniske

**Affiliations:** 1 Faculty of Biology, Genetics, University of Munich (LMU), Martinsried, Germany; Ghent University, Belgium

## Abstract

The Golden Gate (GG) modular assembly approach offers a standardized, inexpensive and reliable way to ligate multiple DNA fragments in a pre-defined order in a single-tube reaction. We developed a GG based toolkit for the flexible construction of binary plasmids for transgene expression in plants. Starting from a common set of modules, such as promoters, protein tags and transcribed regions of interest, synthetic genes are assembled, which can be further combined to multigene constructs. As an example, we created T-DNA constructs encoding multiple fluorescent proteins targeted to distinct cellular compartments (nucleus, cytosol, plastids) and demonstrated simultaneous expression of all genes in *Nicotiana benthamiana, Lotus japonicus* and *Arabidopsis thaliana*.

We assembled an RNA interference (RNAi) module for the construction of intron-spliced hairpin RNA constructs and demonstrated silencing of GFP in *N. benthamiana.* By combination of the silencing construct together with a codon adapted rescue construct into one vector, our system facilitates genetic complementation and thus confirmation of the causative gene responsible for a given RNAi phenotype. As proof of principle, we silenced a destabilized *GFP* gene (*dGFP*) and restored GFP fluorescence by expression of a recoded version of *dGFP*, which was not targeted by the silencing construct.

## Introduction

DNA cloning technologies are instrumental to the functional dissection of biological systems. Recombinant DNA is required to analyze gene and protein function by complementation, localization, overexpression, gene silencing (knockdown) and knockout as well as for the production of proteins or other biomolecules in transgenic organisms. Manipulation and creation of custom DNA sequences is also central in the emerging field of synthetic biology, which aims to engineer new biological components, networks, pathways or even complete organisms for a variety of biotechnological applications [1], [2]. Transgenic plants carrying synthetic DNA sequences are indispensable tools for fundamental research and offer great promise for crop improvements that cannot be achieved by classical breeding [3].

Classical DNA cloning was initiated by the discovery of bacterial type II endonucleases, which in combination with ligases allowed researchers to cleave and rejoin given DNA fragments at restriction sites [4], [5]. By today’s standards this first generation of cloning is relatively time consuming, inflexible, and assembly of large or multiple fragments can be ineffective. For each construct a custom cloning strategy has to be adapted, which is limited by the available restriction sites. Second generation cloning technologies based on homologous site-specific recombination enable very effective high-throughput construction of recombinant DNA and are not dependent on particular restriction sites [6]. Today several commercial and non-commercial systems are available, including Invitrogen’s GATEWAY™ system [7], Clontech’s Creator system, as well as the Univector cloning system developed in the lab of Stephen Elledge [8], [9]. Typically, master or entry vectors are created by insertion of a target PCR sequence (i.e. gene of interest) either by homologous recombination, classical cloning or TOPO cloning (Invitrogen) based on topoisomerases. Once the master clone sequence has been confirmed, the target can be transferred to any compatible expression vector via site-specific recombination. GATEWAY™ cloning in particular was readily adopted in the plant field [10]–[14] as large binary vectors for *Agrobacterium tumefaciens* mediated plant transformations were difficult to handle via classical cloning.

Despite the benefits, current recombination-based cloning approaches are limited in their flexibility. Modular assembly of transcription units (using different promoters, tags, terminators, etc) and construction of plasmids containing multiple genes is difficult to achieve with most recombination systems. MultiSite GATEWAY™ (Invitrogen) partly addresses this need by allowing for the combination of multiple DNA fragments into one expression vector. However, the reagents are not only very expensive, but the number of elements that can be combined in one step is limited to a maximum of four. Furthermore scarless assembly of fragments is not possible as the assembled constructs contain predefined linker sequences. More recently a variety of alternative high-throughput methods have been developed to enable more flexible and effective multi-part DNA assembly. Examples for these include the recombination based in-fusion HD system (Clontech), sequence and ligase independent cloning (SLIC) [15], the Gibson assembly method [16], circular polymerase extension cloning (CPEC) [17] and the seamless ligation cloning extract method (SLiCE) [18]. These systems depend on error prone PCR amplification of the inserts and assembly into linearized backbone vectors, therefore requiring sequence validation for each insert. While being flexible and cost effective compared to commercial options, SLIC, GIBSON, CPEC and SLiCE are limited in that the DNA sequence termini should not contain single stranded secondary structures (as found in many terminators) and that several instances of the same terminal sequence (e.g. present in constructs with identical promoters or terminators) should not be used, as this could lead to loss or rearrangement of individual parts.

These restrictions do not apply to Golden Gate (GG) cloning, which permits the assembly of multiple sequences with high efficiency in one reaction, without the need for PCR amplification or an extra step of vector linearization prior to assembly. GG cloning makes use of type IIS restriction enzymes, which cleave double stranded DNA in a fixed distance from their recognition site to form “sticky end” overhangs [19]–[21] that can be freely defined. By designing overhangs that can anneal in desired combinations, the GG system enables unidirectional assembly of two or more DNA fragments (by placing type IIS sites at their flanking 5′ and 3′ ends). The GG technique has been used successfully in a variety of applications, including assembly of large multigene constructs by iterative approaches [22]–[24] and cloning of designer transcription activator like effectors (TALEs), which are otherwise difficult to assemble because of their repetitive structure [25]–[28].

Based on the GG method we created a cloning toolkit for the modular assembly of *in planta* expression constructs from a set of common modules, including promoters, fluorescence and epitope tags, terminators and *in planta* resistance cassettes. The primary goal of our system was to simplify assembly of multigene constructs. Providing multiple transgenes on the same vector offers a number of benefits, including reliable co-transformation and equal gene dosage and ensures that transcription units are inserted into and thus being influenced by the same chromosomal position. Our toolkit was designed for easy, reliable and flexible construction of vectors for routine cloning applications, such as protein localization, co-immunoprecipitation and gene silencing.

All toolkit vectors and modules (Table S1 and Table S2) are freely available to the public and can be obtained upon request. The annotated plasmid sequences are included in the supplementary data and additionally can be accessed via the JBEI registry (https://public-registry.jbei.org/).

## Results

### Conceptual Design of the GG-based Gene-construct Assembly Tool

With our GG toolkit multiple standardized DNA fragments are assembled in order to create custom T-DNA constructs for gene expression in plants. The system is based on the type IIS restriction enzymes BsaI, BpiI and Esp3I, which generate a 4 bp overhang upon cleavage. Highly effective multi-part assembly is achieved by using a combined restriction and ligation reaction (cut-ligation), whereby re-ligated fragments from given donor plasmids are re-cleaved, while correctly assembled parts are not, as they lack type IIS recognition sites (Figure S1).

**Figure 1:**
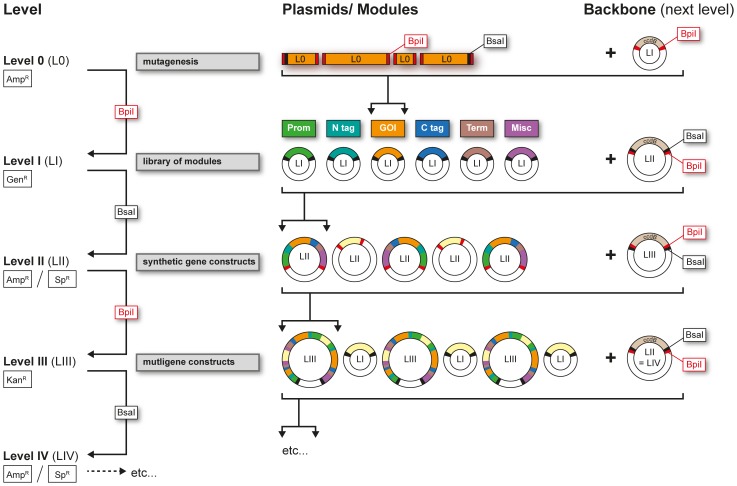
Overview of multi-level construct assembly. The basis for the toolkit is a library of Level I functional modules, consisting of promoters (Prom), amino-terminal tags (N tag), genes of interest (GOI), carboxy-terminal tags (C tag), terminators (Term) as well as miscellaneous modules (Misc). Removal of type IIS sites can be facilitated by creation of L0 fragments for mutagenesis (see Figure S3). Level I modules are assembled into a Level II vector backbone by BsaI cut-ligation to construct synthetic genes. Up to 5 LII synthetic genes are combined into a LIII vector backbone by BpiI cut-ligation to create a LIII binary plasmid. Higher level constructs can be created by sequentially using LII and LIII vector backbones. For instance, LIII multigene assemblies are combined together in a LII backbone to obtain LIV plasmids, while LIV multigene assemblies are again ligated into LIII backbones to create LV constructs. BsaI and BpiI cut-ligations are used in succession to assemble the inserts of one level with the backbone of the next one. Backbone vectors for each level carry a *ccdB* negative selection marker, as well as a different antibiotic resistance, allowing for easy selection of correctly assembled constructs. Level 0 vectors use ampicillin resistance (Amp^R^), Level I vectors gentamicin (Gen^R^), LII cloning-only vectors (Amp^R^), LII binary vectors spectinomycin (Sp^R^) and Level III binary vectors kanamycin (Kan^R^).

A typical construct is assembled in multiple levels (Figure 1): Functional modules (Level I) are combined into a synthetic gene (Level II) with or without added plant resistance maker. Level II (LII) synthetic genes can in turn be assembled into higher-order constructs containing multiple genes (Level III, IV, etc). Each LII construct is assembled from up to six different Level I (LI) modules, namely 1) promoter, 2) amino-terminal tag (N tag), 3) gene of interest (GOI), 4) carboxy-terminal tag (C tag), 5) terminator (Term) and 6) a miscellaneous module (Misc; e.g.: resistance marker). The LI modules are combined with a LII vector backbone by BsaI cut-ligation. Subsequently up to five LII synthetic genes can then be assembled into a single LIII vector backbone by BpiI cut-ligation. For higher order assemblies, the LII and LIII backbones are reused in successive BsaI and BpiI cut-ligations: LIV plasmids are assembled by combining multigene inserts from LIII constructs with a LII backbone using BsaI cut-ligation. In turn, several of these newly derived LIV multigene assemblies can be combined into a LIII backbone to generate a LV plasmid via BpiI cut-ligation. Each vector backbone contains an antibiotic resistance marker different from the previous level (Figure 1), as well as a *ccdB* cassette, which allows for convenient negative selection. The *ccdB* gene product inhibits the *E.coli* DNA gyrase in most lab strains [29], therefore only bacteria containing correctly assembled constructs, which have lost the *ccdB* cassette, are able to grow. The final constructs used for *in planta* transformations are binary plasmids compatible with both *Escherichia coli* and *Agrobacterium*. Two sets of binary plasmid backbones were constructed: one based on the pCambia (Cambia) backbone, the second on pICH50505 (Icon Genetics) (Table S1).

Besides BsaI and BpiI, which are required to sequentially assemble the different construct levels, the toolkit uses Esp3I as a third type IIS enzyme (Figure S1). Esp3I sites enable the assembly of specialized constructs, such as custom backbones for gene silencing, or pre-constructed binary vectors that can be assembled with gene of interest or promoter module in one single BsaI cut-ligation step (see below). All modules and plasmids were cleared of internal BsaI, BpiI and Esp3I sites that would interfere with or reduce efficiency of GG-based construct assembly.

### Level I Module Makeup and Construction

LI modules consisting of promoters, C- and N-terminal tags, GOIs, terminators and miscellaneous elements are flanked by BsaI restriction sites. Upon BsaI cleavage “sticky end” overhangs are created that allow for the assembly of six LI modules into one LII vector backbone. Unique non-palindromic 4 bp sequences (GCGG, TCTG, CACC, AAGG, AATC, TGAG, TGTC) were chosen as appropriate overhangs, which enable the sequential and directional assembly of LI modules. Sequences identical in 3 of the 4 base positions were omitted to avoid potential cross-ligation, caused by the loss of the last base due to exonuclease activity [20]. The GOI overhang sequences (CACC and AAGG) are compatible with a modified pENTR/D-TOPO GATEWAY entry vector (pENTR-BsaI; Table S1), which allows for a combination of GATEWAY technology and GG cloning. GOI LI modules can be inserted into pENTR-BsaI by BsaI cut-ligation and successively cloned into GATEWAY compatible vectors by LR reaction.

**Figure 2:**
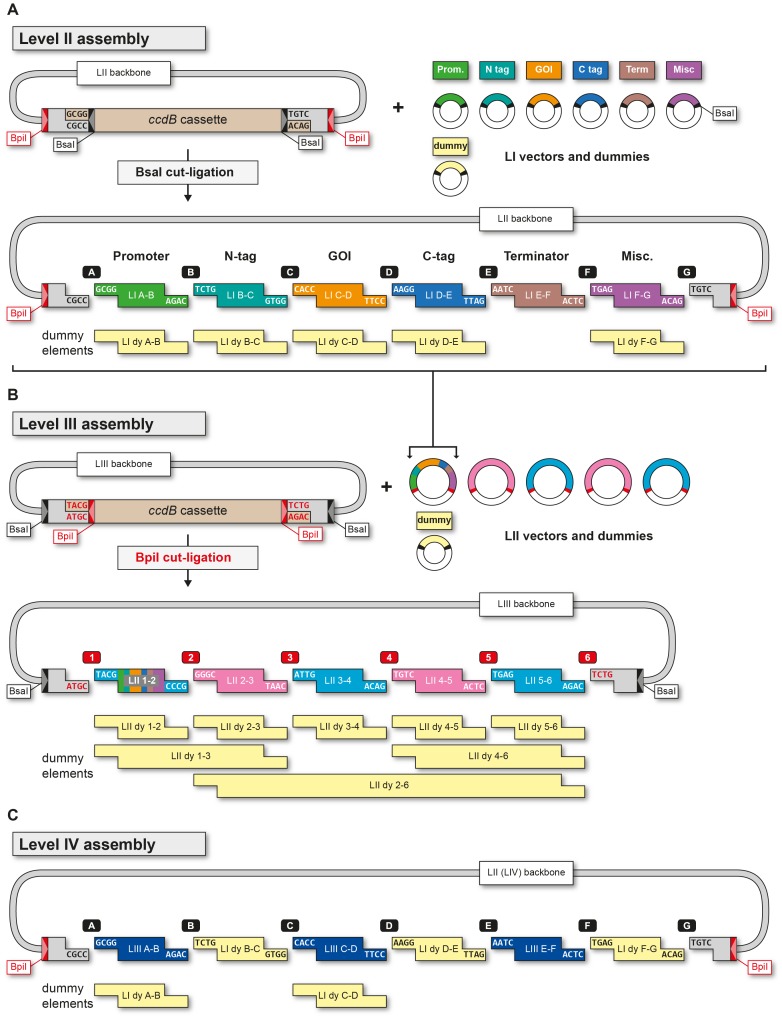
Assembly of Level II, III and IV plasmids. A) LI modules and LI dummies (LI dy) are fused with compatible BsaI overhangs into a LII backbone vector by BsaI cut-ligation. B) To create a LIII binary vector for plant expression up to five LII synthetic genes (LII 1–2, LII 2–3, LII 3–4, LII 4–5 and LII 5–6) or suitable LII dummies (LII dy) are combined with compatible BpiI overhangs into a LIII backbone by BpiI cut-ligation. C) A LIV plasmid is constructed from LIII multigene assemblies combined with LI dy fragments, which carry compatible BsaI overhangs, into a LII backbone by BsaI cut-ligation. LI fusion sites are named A to G, while LII fusion sites are numbered from 1 to 6. Triangles indicate the orientation of the restriction sites relative to the BsaI and BpiI recognition sites.

To create LI modules, target sequences are amplified in a PCR reaction with outer primers containing 5′ BsaI sites and an appropriate terminal extension that results in the desired overhang upon BsaI cleavage (Figure S2). Internal type IIS restriction sites that could interfere with construct assembly (BsaI, BpiI and potentially Esp3I) have to be removed by site-directed mutagenesis. Sequences that do not require removal of type IIS sites can be directly subcloned into a modified pUC57 vector with gentamycin resistance (LI vector backbone) by blunt-end cut-ligation (see methods). To facilitate simultaneous cloning of LI modules and elimination of type IIS restriction sites or for cloning of large constructs from several fragments, we generated the pUC57 based vector LI+BpiI. It contains a *ccdB* negative selection marker flanked by 2 BpiI sites (TACG and TCTG overhangs). For mutagenesis, target sequences are amplified with mutagenic primers (introducing a silent point mutation) containing flanking BpiI sites. Subsequently the PCR fragments are inserted into the LI+BpiI vector by BpiI cut-ligation (Figure S3). The 5′ and 3′ outer primers require an additional inner BsaI site with an appropriate LI overhang, which enables further transfer of the assembled LI module into a LII vector backbone. In our hands, up to four sub-fragments could be ligated directly using column-purified PCR products. To increase the efficiency, the individual fragments are subcloned prior to assembly, since cut-ligations using circular plasmids as donors allow for the combination of at least 9 fragments in one reaction [20]. For subcloning, a standard pUC57 vector (Level 0 vector) with ampicillin resistance is used and fragments are inserted by blunt end cut-ligation. The resulting subcloned inserts are referred to as L0 fragments. For primer design (both with and without mutagenesis), the following additional considerations should be taken into account: A eukaryotic translational initiation sequence (KOZAK) and a start codon are to be introduced into forward primers of N-terminal tag LI modules. As KOZAK sequence we chose the plant consensus AACA(ATG) [30]. Two additional bases need to be added into the reverse primers of N tag- and forward primers of C tag- modules to maintain the frame of the coding sequences. For GOI modules that are to be fused to a C-terminal module, the STOP codon must be removed, to facilitate translational readthrough (Figure S2). The LI overhangs are labeled consecutively from A to G. Plasmids and modules are named according to the flanking overhangs as follows: Promoters “LI A-B”, N tags “LI B-C”, GOIs “LI C-D”, C tags “LI D-E”, terminators “LI E-F” and miscellaneous modules “LI F-G” (Figure 2A). Using this labeling scheme, non-standard modules can also be included, for instance a promoter LI A-C is fused directly to a gene of interest element without the need for an N-terminal tag module.

As part of the LI library we generated a series of fluorescent protein modules, consisting of GFP, T-Sapphire, YFP (Venus), CFP (Cerulean), mOrange and mCherry. Each one is available as N-terminal tag (LI B-C) and as C-terminal tag (LI D-E), and can be used for protein localization and co-localization. The fluorescent tags include a 15 amino acid (aa) flexible linker (3×GGGGS) to minimize interference between tag and the protein of interest. Additionally we generated a GOI version (LI C-D) for each fluorophore both with and without start codon, in order to construct fluorescent transformation markers and tagged fluorophores as co-localization markers. All available LI modules including promoters, terminators, resistance cassettes, small peptide tags and localization signal sequences are shown in Table S2.

### Assembly of Level II, Level III and Higher Level Constructs

Six LI modules are combined into a LII backbone by BsaI cut-ligation to form a LII synthetic gene (Figure 2A). As each LI module produces unique compatible overlaps upon BsaI cleavage, successful assembly into the LII backbone requires the presence of all compatible LI 4 bp overhangs (A to G). In many cases the desired construct does not contain a particular functional module such as an N- or a C-terminal tag. To allow for flexible construction of transcription units, placeholder fragments, called dummies (LI dy), were constructed. A LI dy contains the appropriate BsaI fusion sites to replace a particular LI module. For instance, a LII construct without a C-terminal tag is constructed with a LI dy D-E instead of a LI D-E (C-terminal tag) module. We created LI dummies for each position, except for LI E-F (terminator), which should always be included. When using LI dy A-B and LI dy B-C four additional bases are inserted instead of a LI A-B (promoter) or LI B-C (N-terminal tag) module. Dummies LI dy C-D, LI dy D-E and LI dy F-G contain a short stretch of STOP codons in all frames and both orientations to prevent translational readthrough. Assembled LII plasmids have lost their BsaI sites after successful cut-ligation, however they still contain BpiI sites for the assembly of LIII constructs.

LIII plasmid construction follows the same principle as LII assembly (Figure 2B). Up to five previously created LII synthetic genes are inserted into a LIII backbone in a fixed order and direction by BpiI cut-ligation using unique 4 bp fusion sites (TACG, GGGC, ATTG, TGTC, TGAG, TCTG). The BpiI overhangs are numbered 1 to 6, to distinguish them from the A-G overhangs of the LI modules. Accordingly, the corresponding LII plasmids are named LII 1–2, LII 2–3, LII 3–4, LII 4–5 and LII 5–6. For flexible LIII plasmid construction we also created a series of LII dummy fragments (LII dy), making it possible to assemble LIII constructs by combining 1,2,3,4 or 5 LII synthetic genes. The LII dummies available are LII dy 1–2, LII dy 2–3, LII dy 3–4, LII dy 4–5, LII dy 5–6, LII dy 1–3, LII dy 4–6 and LII dy 2–6 (Figure 2B). We created special dummy versions for positions LII 2–3 and LII 4–5, which besides the stop codons contain 2 repeats of a palindromic 16 bp sequence. These nucleotides have been described to act as an insulator sequence in plants, thus preventing the influence of neighboring promoters on transcriptional activation [31]. The insulator carrying plasmids are named LII ins 2–3 and LII ins 4–5. The orientation of the transcription units in the LIII plasmid can be freely defined, because each LII backbone was created in a forward (F) and reverse (R) version (i.e. LII R 1–2). The LII R vectors have their BsaI fusion sites switched, resulting in the insertion of the LI modules in the opposite direction (Figure S4). If identical promoter and terminator sequences are used, it may be of advantage to orient them in opposite directions pointing towards each other (for instance LII F 1–2 and LII R 3–4). For us, this prevented the rare loss of sequences by homologous recombination, as recombination events in this case cause inversions but not excisions.

Two different sets of LII vector backbones are available: Level II binary plasmids (labeled “LII”), which carry a spectinomycin resistance gene and LII cloning-only plasmids (labeled “LIIc”), which confer resistance to ampicillin. LII binary plasmids contain the *E. coli* origin of replication (ORI) pMB1, the broad host range origin pVS1, as well as the T-DNA left and right border repeat sequences, making them suitable for *Agrobacterium* mediated plant transformations. LIIc plasmids are based on a minimal vector (pAMP) and only contain an *E. coli* ColE1 ORI. They cannot be used directly for plant transformations, but are constructed as intermediates for the assembly of LIII binary plasmids. Typically, cut-ligations into LIIc backbones resulted in around 10 times the number of colonies compared to LII backbones. This higher efficiency can be helpful for large or otherwise problematic constructs. Additionally, LIIc plasmids yielded 3–5 times more DNA in plasmid extractions. Both LII and LIIc plasmids can be freely combined for LIII multigene construct assembly. A list of the available plasmids and dummy modules can be found in Table S1.

LIII plasmids are based on the same binary vector as LII plasmids. However, they contain a bacterial kanamycin resistance gene instead of a bacterial spectinomycin resistance. Besides the BpiI sites needed to combine LII synthetic gene inserts into the LIII vector backbone, LIII vectors contain outer BsaI sites, which allow the fusion of multiple LIII multigene assemblies into a single LIV construct. The LIII BsaI fusion sites fit to the sites present in the LII vector backbones, which in this case act as a LIV vector backbones. We constructed three LIII vector backbones, LIII A-B, LIII C-D and LIII E-F, each available in both forward and reverse orientation. The LIII multigene assemblies can be combined with LI dummies LI dy B-C, LI dy D-E, LI dy F-G and a LII vector backbone by BsaI cut-ligation to create a LIV construct (Figure 2C). This allows for a current maximum assembly of 15 synthetic genes into a LIV plasmid. Higher level constructs are created in the same fashion, for instance LIV multigene assemblies can be combined into a LIII backbone to form LV constructs by BpiI cut ligation.

We found our toolkit to work with high precision for both single and multigene assembly. Fidelity of the constructs was determined by restriction digestion followed by sequencing of the fusion sites. In a total of 105 assembled LII constructs and 210 isolated plasmids we found only a single assembly error. For 110 multigene constructs we isolated plasmids from 220 bacterial colonies and did not detect any errors, resulting in a combined cloning accuracy of over 99%.

### Creation of Preassembled Vector Backbones for the Targeted Insertion of Single Modules

**Figure 3:**
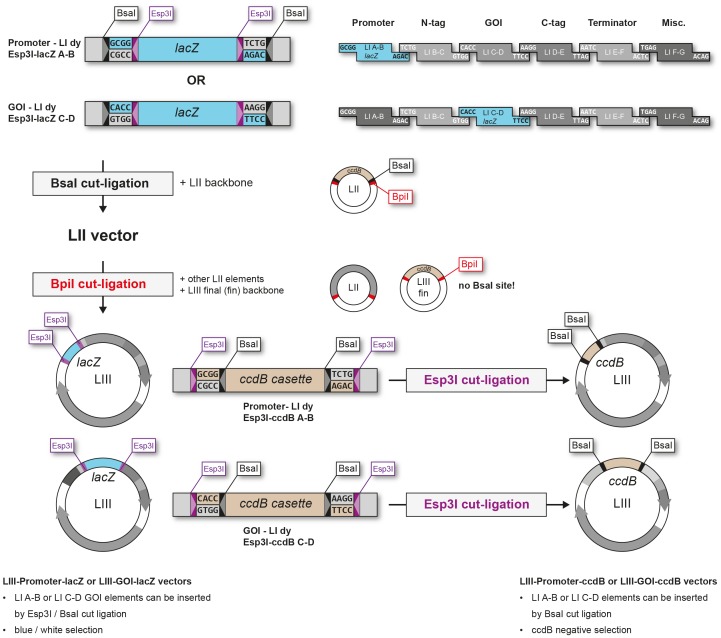
Construction of custom binary vector backbones for promoter and gene of interest analysis. Pre-assembled binary constructs are created with Esp3I-lacZ dummies. The desired LI modules are assembled together with a LI dy-Esp3I-lacZ A-B fragment instead of a specific LI A-B promoter module or a LI dy-Esp3I-lacZ C-D fragment instead of a specific LI C-D GOI module into a LII backbone by BsaI cut-ligation. The resulting LII assembly is combined with inserts from other LII constructs into the LIII final (LIII fin) backbone by BpiI cut-ligation. Into the resulting LIII binary vectors LI A-B or LI C-D modules can be directly inserted by a combined BsaI+Esp3I cut-ligation. Alternatively the LIII binary vectors can be further refined in a second step, by addition of a *ccdB* dummy fragment (LI dy-Esp3I-ccdB C-D) via Esp3I cut-ligation. The final version of the pre-assembled LIII binary vector backbone can be combined with custom LI A-B or LI C-D modules in one BsaI cut-ligation using *ccdB* based negative selection.

Although efficiency and accuracy in GG-based assembly is high, construction of binary plasmids containing multiple genes still requires several steps. In a certain experimental setup (e.g. screening of different promoters or genes) it may be desirable to only exchange a single element, but keep the remaining modules constant. For this type of experiment we created an extension to our toolkit, in which a custom backbone can be established that upon validation can be used in a two-component ligation to insert individual promoters (LI A-B) or genes (LI C-D). First, the backbones are assembled analogous to other binary constructs for plant expression by repeated BsaI and BpiI cut-ligations. However, instead of a promoter or GOI LI module, special dummies are inserted, which contain a *lacZ* gene fragment flanked by cleavage sites for the type IIS enzyme Esp3I (LI dy Esp3I-lacZ A-B for promoters and LI dy Esp3I-lacZ C-D for GOIs). The Esp3I sites are arranged in a way to create the same overhang as the LI BsaI sites (Figure 3). Once the newly constructed backbone is sequence-validated it can be combined with the desired functional modules (e.g. variations of a promoter) by a mixed cut-ligation using both BsaI and Esp3I enzymes, thus replacing the corresponding Esp3I dummy fragment. Positive clones can be identified by blue/white screening on plates containing X-Gal and IPTG (positive clones have lost the *lacZ* gene fragment and appear white). The custom vector backbone can be further refined in a second step by replacing Esp3I sites with BsaI sites and the *lacZ* selection with the *ccdB* gene, which allows for an easier and more reliable selection without the need for any supplements, such as X-Gal, to the growth medium. The exchange is achieved by insertion of additional dummy fragments, which contain a *ccdB* cassette (*ccdB* gene and chloramphenicol resistance) flanked by Esp3I and BsaI sites (LI dy Esp3I-ccdB A-B or LI dy-Esp3I-ccdB C-D). The Esp3I and BsaI sites are oriented in such a way that following Esp3I cut-ligation the custom backbone is left with only BsaI sites and the appropriate overhangs for insertion of LI A-B or LI C-D modules (Figure 3). Positive backbone clones are screened for by selection against the chloramphenicol resistance marker and the resistance of the vector backbone (kanamycin for LIII, spectinomycin for LIV). LI A-B or LI C-D modules can be inserted into the second version of the custom build vector (i.e. LIII-GOI-ccdB) by a single BsaI cut-ligation. For custom vector construction, we created an additional LIII backbone, which does not contain BsaI sites, as these would interfere with the insertion of LI modules by BsaI cut-ligation. This LIII backbone was named LIII final (LIII fin) and can also serve as a LV backbone. Standard LIV ( = LII) backbones can be used without further modifications, since a completely assembled LIV custom vector does not contain any more BsaI sites.

### Golden Gate Based Gene Silencing Kit

**Figure 4:**
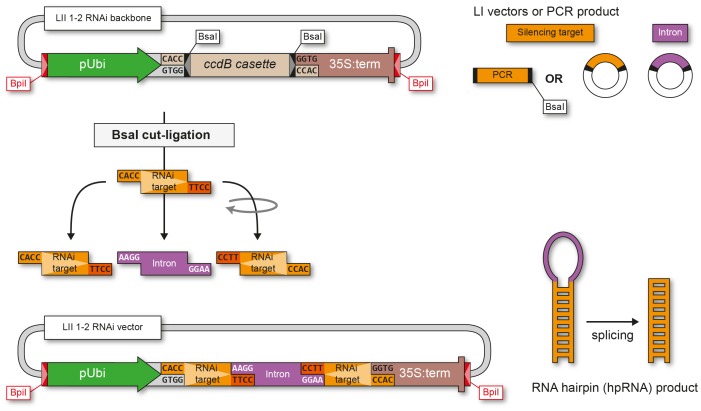
Construction of plasmids for RNAi mediated gene silencing. A) A target sequence containing LI C-D fusion sites and a LI intron element are combined into a LII 1–2 RNAi vector backbone by BsaI cut-ligation. The chosen BsaI overhangs enable a one-step assembly of a hairpin construct containing the intron element between two copies of the LI C-D target sequence. When expressed, this creates an intron-spliced hairpin RNA, which is effective in gene silencing.

As an addition to our toolkit, we provide a GG based system for RNAi mediated gene silencing. Custom RNAi vectors (LII F 1–2 RNAi) are used to assemble silencing constructs that express intron-spliced hairpin (ihp) RNAs, which are able to induce strong silencing [32]. The LII F 1–2 RNAi vector backbones were constructed from standard LI modules and custom Esp3I dummies and contain the *L. japonicus* ubiquitin promoter, a *ccdB* cassette for negative selection flanked by compatible BsaI overhangs for insertion of LI C-D modules and a 35S terminator (Figure S5). The silencing backbones are available as an intermediate cloning-only version (LIIc) and as a binary plasmid suitable for *Agrobacterium* mediated transformation (LII). Target sequences for silencing are amplified by PCR with primers containing LI C-D BsaI fusion sites. Alternatively a LI C-D module can be integrated directly as a target. For RNAi construct assembly, the LII F1–2 RNAi backbone is combined with the target LI C-D module and a LI plasmid containing an intron element (intron1 of *AtWRKY33* [*33*]). BsaI sites of the backbone and the intron were chosen in a way that two copies of the LI C-D module are assembled in opposite direction separated by the intron, thus forming an intron-spliced hairpin RNA construct in a single BsaI cut-ligation (Figure 4).

### Binary Plasmid Assembly and Plant Transformation – proof of Principle

#### Construction of multigene binary plasmids

**Figure 5:**
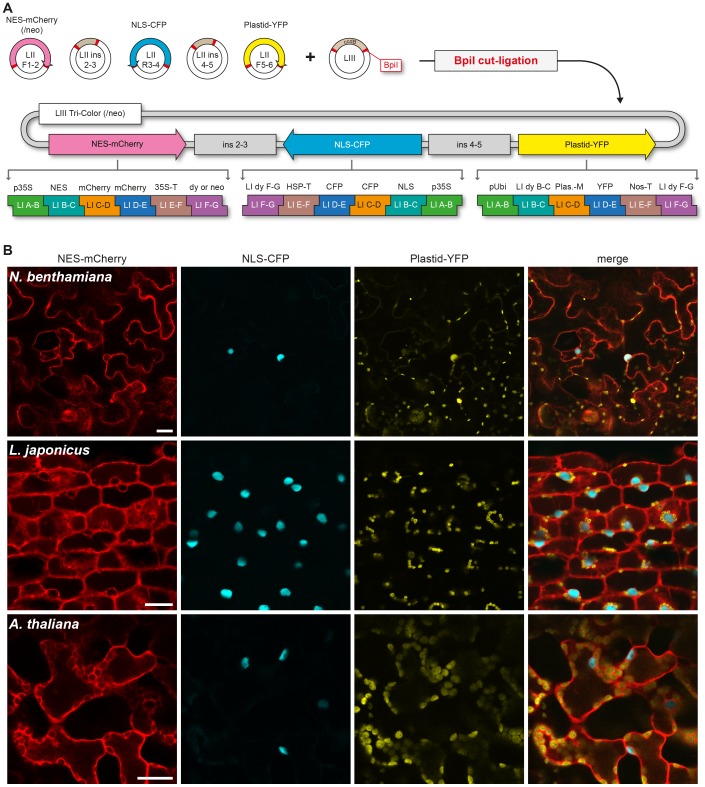
*In planta* expression of LIII constructs. A) Binary plasmids LIII Tri-Color and LIII Tri-Color (neo) were constructed from LII F 1–2 NES-mCherry or LII F1–2 NES-mCherry (neo), LII R 3–4 NLS-CFP, LII F 5–6 Plastid-YFP assemblies and LII insulator fragments LII ins 2–3 and LII ins 4–5 by BpiI cut-ligation into a LIII vector backbone. All LI modules contained in the final LIII construct are depicted under the plasmid map. B) Confocal laser scanning microscopic (CLSM) images of transformed plants. LIII Tri-Color was expressed in *N. benthamiana* leaves and *L. japonicus* roots by *Agrobacterium* mediated transformation. LIII Tri-Color (neo) was used to generate stable transgenic *A. thaliana* lines. p35S = cauliflower mosaic virus 35S promoter; pUbi = *L. japonicus* polyubiquitin promoter; NES = nuclear export signal; NLS = nuclear localization signal; 35S-T = cauliflower mosaic virus 35S terminator; HSP-T = heat shock protein terminator of *A. thaliana*; nos-T = nopaline synthase terminator; neo = neomycin resistance cassette; Plas-M. = Plastid Marker (plastid localized protein of *L. japonicus*). Scale bars = 25 µm.

To demonstrate usability and versatility of our system we assembled binary plasmids with multiple transcriptional units and tested expression in transiently transformed *Nicotiana benthamiana* leaves, transgenic *Lotus japonicus* roots (hairy roots) and stable transgenic *Arabidopsis thaliana* plants. The construct used for *A. thaliana* transformation included an additional neomycin resistance gene (LI F-G module) to facilitate selection of transformed plants. The final LIII plasmids were named LIII Tri-Color and LIII Tri-Color/neo. The plasmids contained transcriptional units for co-expression of three different fluorophores (CFP, YFP and mCherry) in distinct cellular compartments (nucleus, cytosol, plastid/chloroplast). mCherry was tagged with a nuclear export signal (NES), CFP with a nuclear localization signal (NLS) and YFP was fused to a plastid localized protein of *L. japonicus*. Both mCherry and CFP were used in tandem to increase the size of the resulting fusion product, thus preventing passive diffusion through the nuclear pore. A detailed list of the LI modules and LII vectors used for LIII plasmid assembly is shown in Figure 5A. *N. benthamiana* leaves, *L. japonicus* roots and *A. thaliana* leaves and roots transformed with the LIII constructs showed strong co-expression of the three fluorophores with distinct localization patterns (Figure 5B). The NLS tagged CFP showed exclusive nuclear localization, while the NES tagged mCherry was only visible in the cytosol. The YFP-tagged plastid protein of *L. japonicus* depicted the expected plastid localization in *L. japonicus* roots and chloroplast localization in *A. thaliana* leaves, while in *N. benthamiana* besides a signal in chloroplasts additional nuclear and cytosolic localization could be observed. The latter observation is potentially an artifact caused by the very high expression of the heterologous protein in the *N. benthamiana* system. In sum, transfection of the generated multigene construct showed the expected localization with high expression levels in different plant species, thereby demonstrating the functionality of the GG toolkit.

#### Assembly of custom binary vector backbones

Using the GOI Esp3I dummies (LI dy Esp3I-lacZ C-D and LI dy-Esp3I-ccdB C-D) we constructed a custom LIII vector backbone for protein localization. The backbone was assembled with the following transcription units: 1) A ubiquitin-promoter with the GOI-BsaI-ccdB cassette and a C-terminal YFP-Venus fusion. 2) mCherry driven by a 35S promoter as a visual transformation marker. 3) A 2x-CFP with an N-terminal NLS under a 35S promoter as a marker for nuclear localization. The final version of the vector contained LI C-D BsaI sites and a *ccdB* cassette (LIII GOI-YFP; Figure S6). To validate functionality of the backbone we inserted the genomic sequence of the *L. japonicus* plastid protein (LI C-D Plastid-Marker) that we had used previously (Figure 5), by BsaI cut-ligation. All analyzed clones of the custom vector backbones containing the dummy modules (16 in total) and of the final LIII Plastid-YFP binary plasmid (8) were correctly assembled without errors. Upon *Agrobacterium* mediated transformation of the binary construct (LIII Plastid-YFP) we observed the expected subcellular localization: cytoplasmic mCherry, nuclear localized CFP and YFP localized to plastids, nucleus and partly to the cytosol (Figure S6).

#### Assembly of gene silencing and silencing rescue constructs

**Figure 6:**
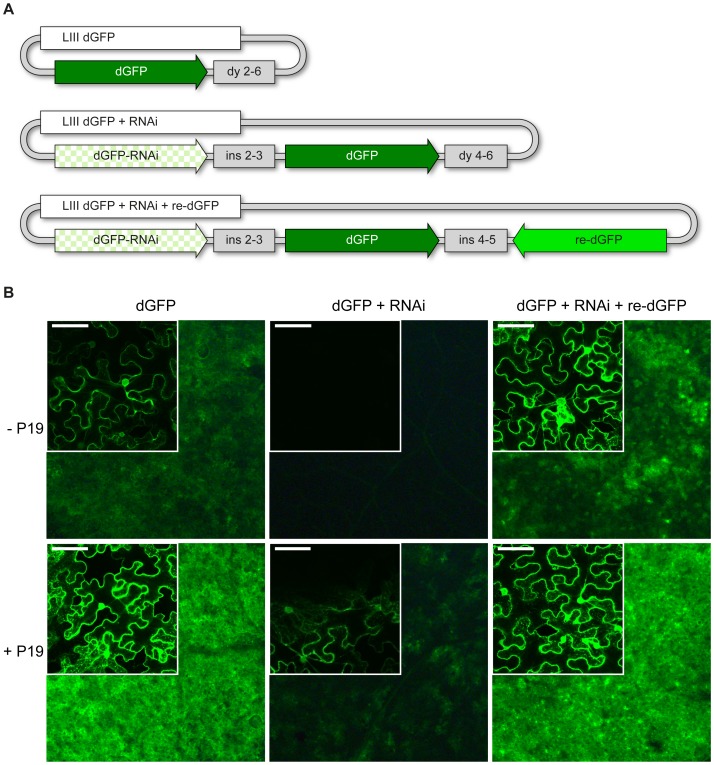
GFP silencing and complementation. A) Schematic representation of plasmids LIII dGFP, LIII dGFP+RNAi and LIII dGFP+RNAi+re-dGFP. B) Expression of destabilized GFP (dGFP) was assayed in *N. benthamiana* two days after *A. tumefaciens* mediated transformation. The larger stereomicroscopic pictures depict 5×5 mm^2^ leaf sections, while the inlay pictures show confocal scans of single epidermal cells (scale bar = 75 nm). For image acquisition the same settings were used for all samples. Co-expression of dGFP together with the RNAi construct targeting *dGFP* mRNA completely silences dGFP expression. A recoded version of *dGFP* (*re-dGFP*) is immune to silencing and able to restore strong dGFP fluorescence despite expression of the RNAi construct. Addition of P19 is able to suppress dGFP silencing, but only partly: expression of dGFP together with the RNAi construct is weaker and spottier than expression of dGFP alone.

To demonstrate successful silencing we targeted a GFP transgene. A LIIc F 1-2 GFP-RNAi plasmid was assembled with a pre-existing LI C-D GFP module, thereby creating a hairpin construct with the full-length sequence of the gene. Among 16 colonies tested we did not detect any errors in the assembly of the hairpin construct. Due to the symmetric overhangs used for hairpin formation, the intron sequence of the RNAi vector was able to integrate in either orientation during cloning (Figure 6) with about 50% likelihood (7 of 16 clones contained an inverted intron). We selected LII clones with a correctly oriented intron sequence, as only these would be spliced out, while the inverted intron would only act as a spacer. Two LIII binary plasmids were assembled: 1) The LIIc F 1–2 GFP-RNAi assembly was combined with a LIIc F 3–4 GFP assembly and a LIII backbone to obtain the silencing construct LIII GFP+RNAi; 2) A control plasmid LIII GFP was constructed only with a LII 3–4 GFP insert (Figure S7). We transformed *N. benthamiana* leaves with the silencing construct and the GFP control plasmids. Expression of the LIII GFP-only construct showed strong fluorescence, which was absent in leaves infiltrated with the LIII GFP-RNAi binary vector, indicating successful silencing (Figure S7).

Off-target effects in post-transcriptional gene silencing by siRNAs can cause phenotypic side effects. To determine the specificity of the silencing phenotype the observed mutant phenotype can be rescued by a codon-modified target gene. With our toolkit a LII RNAi assembly can be combined with another LII synthetic gene containing a recoded version of the target sequence, to test for successful complementation. This recoded gene variant shows low nucleotide similarity to the target gene but translates into the same protein. Since gene silencing specificity is determined by nucleotide and not protein homology, the recoded variant is not affected by silencing of the target gene. As proof of principle for silencing rescue, we generated a binary plasmid (LIII) that contained both a silencing construct targeting destabilized *GFP* (*dGFP*) as well as a recoded version of *dGFP* (*re-dGFP*) that was obtained by gene synthesis. To generate the LII silencing construct the first 480 bp of *dGFP* were amplified with primers containing LI C-D BsaI sites and directly integrated into the LII F 1–2 RNAi backbone vector by BsaI cut-ligation. The *re-dGFP* sequence was derived from *dGFP* by introducing a maximum number of silent codon exchanges into the sequence, using the GeneDesign3.0 codon juggling algorithm [34]. The resulting sequence was further codon adapted for *in planta* use by the jCat tool [35] and then manually edited to prevent sequence identical regions longer than 5 bp compared to the original *dGFP* (Figure S8). To assess silencing and rescue we constructed three LIII binary plasmids: 1) LIII dGFP, which expressed only destabilized GFP. 2) LIII dGFP+RNAi, which expressed both dGFP as well as the silencing construct targeting the first 480 bp of the gene. 3) LIII dGFP+RNAi+re-dGFP, which expressed dGFP, the silencing construct and the recoded re-dGFP. The constructs were expressed in wild type *N. benthamiana* leaves and GFP fluorescence was analyzed two days after *Agrobacterium* mediated transformation. Transfection of the LIII dGFP construct resulted in the expected expression of the fluorophore throughout the infiltrated leaf. Leaves that were infiltrated with the LIII dGFP+RNAi plasmid were devoid of any GFP signal, indicating efficient *GFP* silencing. By contrast, the triple construct containing the recoded *re-dGFP* gene again showed strong fluorescence, suggesting that *re-dGFP* mRNA is not targeted by the *dGFP*-silencing construct (Figure 6). Fluorescence of re-dGFP was increased compared to dGFP, likely due to the plant adapted codon usage of the gene construct. As a control, we co-infiltrated each of the constructs in combination with an *A. tumefaciens* strain mediating expression of the viral silencing suppressor P19, which binds siRNA with high affinity and thus prevents its incorporation into the RNA-induced silencing complex (RISC) [36], [37]. As expected, both dGFP and re-dGFP showed stronger fluorescence in the presence of P19. Unlike for LIII dGFP+RNAi alone, leaves infiltrated with LIII dGFP+RNAi and the P19 vector showed dGFP signal, which however was spotty and weaker than in leaves infiltrated with the P19 construct and LIII dGFP. This indicates that RNAi mediated gene silencing is only partially suppressed by P19 in leaves expressing dGFP+RNAi.

## Discussion

### Reliable and Flexible Assembly of Customized Binary Plasmids

Inexpensive, efficient and reliable assembly of multiple fragments is the hallmark of GG cloning [19]–[21]. We established a GG toolkit for the construction of binary plasmids for single and multigene expression in plants. To validate the functionality of the system we generated localization and silencing constructs containing multiple transcription units and demonstrated successful transgene expression in *N. benthamiana*, *L. japonicus* and *A. thaliana*.

One major goal of our toolkit was to simplify the assembly of binary plasmids containing multiple genes. The system makes use of the type IIS enzymes BsaI and BpiI to iterativly construct binary constructs from a common set of functional modules (promoters, amino- and carboxyterminal tags, genes of interest, terminators and resistance casettes). High flexibility in combination with the high cloning efficiency make it possible to assemble variants of one construct with minimal effort, once the individual modules are present in the library. As our plasmids have shown high-level expression in different plant species, the constructs can be pre-screened by transient *N. benthamiana* transformation and then directly be used for generation of stable lines or transgenic (hairy) roots.

Due to the presence of predefined fusion sites and the use of dummy fragments, short foreign sequences (scars) are introduced when assembling transcriptional units. In case of the N-terminal dummy (LI dy B-C) 4 bases are added in front of the CACC fusion site sequence (which also serves as a KOZAK sequence), while the C-terminal dummy (LI dy D-E) causes the addition of 2 amino acids into the reading frame before introducing a stop codon. These additions are shorter than in GATEWAY assembly, which leaves scars of 21 bases. However, for critical applications it is possible to circumvent any alterations to the sequence by creation of non-standard LI modules or unique overhangs. For example, a complementation construct can be constructed as a single LI A-F module consisting of native promoter, gene, and native terminator, without addition of bases in the promoter region or any changes in the amino acid sequence. The LI A-F module can then be combined with a LI F-G *in planta* resistance and further assembled into a LII and a LIII vector together with other desired transcription units by standard BsaI and BpiI cut-ligations.

Translational readthrough, transactivation by neighboring promoters and unintended recombination events are potential concerns when assembling multigene constructs. We included additional STOP codons in all frames and orientations in LI dummies and LII dummies, in order to minimize the possibility of translational readthrough. To add additional flexibility in construct design, we constructed a “reverse” version for each LII and LIII backbone vector, which allows for the inserted sequence to be flipped around and thus freely define the orientation of each transcription unit. Unintended transcriptional readthrough, as well as transactivation by neighboring constructs could potentially be reduced or prevented by changing the orientation of LII or LIII constructs.

### Toolkit Customization and Extension

By default, our LII and LIII binary vectors contain only sequence elements required for construct assembly (type IIS restriction sites) and plant transformation (T-DNA borders). Transformation markers are not part of the vector backbones to keep these as flexible as possible. LII and LIII vectors were constructed from precursors, which contain Esp3I sites inside of the T-DNA border sequences, but no BsaI or BpiI sites (Table S1). These precursors can be adapted to construct new and customized LII or LIII vector backbones by addition of fragments with appropriate fusion sites via Esp3I cut-ligations.

Esp3I cut-ligations can also be used to create custom stand-alone GG based vector backbones for gene of interest or promoter screens. For that LII, LIII or higher level plasmids are created with placeholder modules, which can later be replaced by individual promoters or genes, without the need to assemble the complete construct again. The custom build vectors can also be used independently from the rest of the system. An added advantage of these backbones is that for cloning of given inserts only one type of type IIS recognition site (either BsaI or BpiI) has to be removed.

### Golden Gate Based Gene Silencing and Rescue

Gene silencing in plants and animals is a popular reverse genetics tool to study gene function. As a toolkit addition we created a silencing vector for intron-containing hairpin RNA (ihpRNA) constructs and demonstrated silencing of *GFP* in *N. benthamiana*. GG based silencing vectors have been described previously [38], however the integration of a LII silencing construct into our toolkit offers several advantages over a stand-alone plasmid. Silencing constructs targeting several genes at once can be created by combining multiple target sequences into the LI+BpiI vector, which can then be directly integrated into a LII RNAi construct. As the LII silencing backbone itself was constructed using the GG toolkit, it can be fully customized (e.g. by inclusion of an *in planta* resistance gene) or further assembled into higher-level constructs.

Complementation of mutant phenotypes is a standard requirement when studying gene functions. However, often the same stringency is not applied to RNAi based analyses, even though off-target effects due to sequence similarity to other genes can potentially result in phenotypes, which are not related to the target gene. Genome-wide profiling has shown that potentially dozens of transcripts (even with limited sequence similarity) can potentially be affected by silencing [39], [40]. To validate that an observed phenotype is not caused by unintended down-regulation at off-target sites, the silencing phenotype can be rescued by expression of a codon-modified version of the target gene [41]. Our system allows the easy assembly of such a control, by combining a LII silencing assembly together with a LII assembly containing a recoded version of the target gene that can be obtained by gene synthesis. We demonstrated this by knock-down of *dGFP* and complementation with a recoded *dGFP* version that was not recognized as a silencing substrate and therefore remained fully active. The same principle can be applied to any RNAi targeted gene, in order to rescue an observed phenotype and thus confirm its specificity. As both silencing and complementation construct are part of the same binary plasmid, there is a high chance of co-expression throughout the transformed tissue. Therefore, stable lines for silencing and complementation can be generated in parallel and do not require two sequential transformation steps.

### Alternative GG Based Systems

Recently two other GG based assembly systems termed “modular cloning” (MoClo) [22] and Goldenbraid/Goldenbraid 2.0 [23], [24], have been developed. Compared to our toolkit, both systems offer different kinds of modules and assembly strategies. For instance, Goldenbraid 2.0 defines 11 standard parts that form a transcriptional unit, which can be arranged into larger superclasses [24] and uses both multipartite as well as binary assemblies. As both systems do not use dummy fragments for assembly of synthetic genes, they require variants of each module with different fusion sites to allow for flexible assembly (e.g. different promoter versions are needed to assemble a transcription unit with or without an N-terminal tag), which overall increases the number of toolkit parts significantly. The higher number of modules and combinatorial possibilities ultimately allows for more fine control and minimizes potential scars, however it also makes these toolkits more complex. As GG based systems are finding more widespread adoption, the overall number of modules that have been freed of common type IIS restriction sites continuously increases [22]–[24], [42]. While the chosen overhangs are often not directly compatible, it is relatively easy to convert type IIS liberated modules to a particular system by a single PCR reaction with outer primers providing the appropriate fusion sites.

## Materials and Methods

### Plasmid Construction

A list of primers and templates used for the construction of modules and plasmids is provided in Table S3. Binary plasmids were created based on two different backbones, LIIα/LIIIα plasmids originate from pICH50505 (iCON Genetics) and LIIβ/LIIIβ from pUB-GW-HYG [33] (CAMBIA). The experiments presented in the manuscript were done using LIIα/LIIIα backbones, which are therefore referred to as LII or LIII only. For binary plasmid construction, BsaI, BpiI and Esp3I sites as well as all backbone sequences except for the replication origins and *Agrobacterium* left and right border sequences were removed and two new Esp3I sites were inserted between the left and right border sequences. The original resistance genes were replaced with a kanamycin resistance gene amplified from pENTR/D-TOPO (Invitrogen) for LIII backbones (precursors Xpre-K and Xpre2-K) and with a spectinomycin resistance amplified from pK7WG2D [43] for LII backbones (precursors Xpre-S and Xpre2-S). Into the precursor constructs *ccdB* cassettes (*ccdB* and chloramphenicol resistance gene) were added between the borders by Esp3I cut-ligation, flanked by appropriate type IIS fusion sites.

### Molecular Biology Reagents and Techniques

Restriction enzymes were obtained from New England Biolabs (NEB) and Thermo Scientific/Fermentas. T4 Ligase was obtained from NEB. Plasmids were prepared using the Thermo Scientific GeneJET Plasmid Miniprep Kit. PCRs were performed with the Phusion®High-Fidelity Polymerase (NEB). PCR products were cleaned up with the GeneJET PCR Purification Kit (Thermo Scientific).

### Biological Material, Growth Conditions and Plant Transformation

For cloning *Escherichia coli* Top10 and DB3.1 (for fragments containing *ccdB*) were used. Strains were grown in LB medium at 37°C (200 rpm) with kanamycin, carbenicillin (instead of ampicillin) at 50 µg/ml, spectinomycin at 100 µg/ml and gentamycin at 10 µg/ml. *Agrobacterium tumefaciens* AGL1 [44] was used for *N. benthamiana* and *A. thaliana* transformation, *A. rhizogenes AR1193* [45] for *L. japonicus* transformation. *Agrobacterium* strains were grown at 28°C (200 rpm) in LB medium with 20–50 µg/ml kanamycin +50 µg/ml carbenicillin or 100 µg/ml spectinomycin +50 µg/ml carbenicillin. *Lotus japonicus* ecotype B-129 Gifu was used as wild type. *L. japonicus* hairy root plants were generated as previously described [46]. Stable transformation of *A. thaliana* was performed by floral dip [47] and transformation of *N. benthamiana* leaves was done as previously described [36]. Stable transformants of *A. thaliana* were identified by selection on half-strength MS medium [48] containing 50 µg/ml kanamycin.

### Cloning Protocols

#### BsaI, BpiI and Esp3I cut-ligation

For convenience all plasmids were diluted to a final concentration of 100 ng/µl. For BsaI, BpiI (BbsI) and Esp3I (BsmBI) cut-ligations 1 µl of each plasmid was combined in a 15 µl reaction together with 0.5 µl of enzyme (5–10 units), 0.75 µl T4 ligase (NEB) and 1.5 µl ligase buffer. For BsaI cut-ligations 0.15 µl of bovine serum albumin (10 mg/ml) were added. Reactions were incubated in a thermocycler for 20–40 cycles, cycling between 37°C for 2 min and 16°C for 5 min, followed by 37°C for 5 min, 50°C for 5 mins and 80°C for 5 mins. 3–5 µl of the reaction were transformed into *E. coli* TOP10 or DB3.1 (for insertion of *ccdB* cassettes).

#### Blunt-end cut-ligation

For blunt-end cloning of PCR fragments into the multiple cloning site of pUC57 vectors an optimized cut-ligation protocol was used. The vector was combined with insert in a molar ratio of 1∶5 to 1∶10. For a 20 µl reaction 0.4 µl of restriction enzyme (5–10 units), 1 µl T4 ligase (NEB) and 2 µl ligase Buffer were added. Typical blunt cutters used were: NruI, StuI and SmaI (NEB). For SmaI, addition of 2 µl NEB restriction buffer 4 was required. The reaction was incubated in a thermocycler for 20–30 cycles, cycling between 37°C for 5 min (20°C for SmaI) and 20°C for 5 min, followed by heat inactivation at 80°C for 10 min. 0.4 µl of fresh enzyme was added together with 0.4 µl of antarctic phosphatase (NEB) and 2 µl of phosphatase reaction buffer. Samples were incubated for another 30 min at 37°C (20°C for SmaI), then heat inactivated at 80°C for 20 min. 5 µl of the reaction was transformed into *E. coli* Top10 by heat shock and plated on LB plates containing the appropriate antibiotic supplemented with 40 µg/ml X-Gal (5-Brom-4-chlor-3-indoxyl-β-D-galactopyranosid) and 100 µM IPTG (Isopropyl β-D-thiogalactopyranoside). Plasmids were isolated from white colonies and validated by sequencing.

### Microscopy

Confocal Laser-Scanning Microscopy (CLSM) of transformed *N. benthamiana* leaves, *L. japonicus* roots and *A. thaliana* leaves was performed with an upright Leica SP5 confocal laser scanning microscope. *L. japonicus* hairy roots were kept under a gas permeable plastic film (lumox®Film) and imaged using a long-distance Leica HCX IRAPO L 25×/0.95 W objective. *N. benthamiana* and *A. thaliana* leaves were vacuum infiltrated prior to imaging and imaged with a HCX PL Fluotar 20×/0.5 PM2 objective. For image acquisition the resolution was set to 512×512 or 1024×1024 pixels and the frame average to 4. Using the argon laser at 20% power, GFP was excited with the 488 nm laser line and detected at 500–530 nm, CFP was excited with the 458 nm spectral line and detected at 465–505 nm, YFP with the 514 nm spectral line and detected at 530–550 nm. mCherry was excited with a diode-pumped solid state (DPSS) Laser at 561 nm and detected at 580–620 nm. For multi-color imaging the frame sequential scan mode was used. Overview pictures of *N. benthamiana* leaves were taken using a Leica M165 FC epifluorescence stereomicroscope with a GFP filter.

## Supporting Information

Figure S1 **Type IIS restriction enzymes and Golden Gate cut-ligation.** A) Recognition and restriction sites of type IIS endonucleases BsaI, BpiI and Esp3I. B) Cut-ligations combine restriction and ligation in one single cyclical reaction, allowing for efficient assembly of multiple fragments and continuous cleavage of undesired starting products.
(TIF)

Figure S2 **LI modules and primer design for amplification of template sequences without mutagenesis.** A) Classes of LI modules with their respective overhangs after BsaI cleavage. B) Outer sequence of the forward (Fw.) and reverse primers (Rv.) used to amplify a particular LI module without mutagenesis. As blunt end subcloning sometimes resulted in the loss of the terminal 5′ bases, 2 additional bases (NN) were included at the 5′ position of the primers, in order to increase the number of correct clones.
(TIF)

Figure S3 **Generation of LI modules with removal of type IIS sites.** A) A target sequence with undesired restriction sites is amplified in separate PCR reactions with mutagenic primers containing BpiI sites. Assembly of the mutagenized fragments is done by BpiI cut ligation into the LI+BpiI vector backbone, either directly from PCR fragments or after blunt-end subcloning into a L0 vector. B) Details on primer design and mutagenesis. The outer primers contain an additional BsaI site, which is required to assemble the finished LI module into a LII vector. The inner primers introduce a silent mutation into the target sequence in order to remove the original type IIS restriction site. For details on the specific LI element overhangs as well as additional consideration for primer design see Figure S2.
(TIF)

Figure S4 **LII vector backbones define the orientation of the transcription units in LIII constructs.** A) LII backbones vectors are available in a forward (F) and reverse (R) version. In the LII R backbone BsaI overhangs are flipped, thus resulting in the insertion of the LI modules in inverse orientation after BsaI cut-ligation. B) Exemplary construction of LII assemblies in different orientations into a LIII vector backbone.
(TIF)

Figure S5 **Vector backbone construction for RNAi mediated gene silencing.** First a preliminary construct (LII F 1–2 RNAi-pre) was assembled out of a LII F 1–2 backbone, a ubiquitin promoter (pUbi) module (LI A-B), a 35S-terminator (LI E-F), a LI dy F-G and a custom dummy (LI B-E Esp3I dy) module by BsaI cut-ligation. The LI B-E Esp3I dy contains BsaI sites which fit to the B and E overhangs of LI modules as well as inner Esp3I sites which generate the same B and E overhangs when cleaved. In a second step a *ccdB* cassette containing matching Esp3I and additional inner BsaI sites was combined with the LII F 1–2 RNAi-pre vector by Esp3I cut-ligation. The finished LII F 1–2 RNAi vector contains the *ccdB* cassette flanked by BsaI sites with the fusion sites CACC and GGTG, which can be used to insert 2 copies of a LI C-D element together with an intron element.
(TIF)

Figure S6 **Expression of mCherry, CFP and YFP using a custom LIII binary vector.** A) The LIII Plastid-YFP binary plasmid was constructed by insertion of a LI C-D Plastid-Marker (genomic sequence of plastid localized protein from *L. japonicus*) module via BsaI cut-ligation into the LIII-GOI-YFP vector backbone, which was preassembled with free mCherry and NLS-2xCFP fluorescence markers B) CLSM images of *N. benthamiana* transformed with the LIII Plastid-YFP construct 2 days after *Agrobacterium* mediated transformation. Scale bar = 25 µm.
(TIF)

Figure S7 **RNAi mediated knock-down of GFP.** A) Schematic representation of LIII binary constructs LIII GFP and LIII GFP+RNAi. B) CLSM images of *N. benthamiana* leaves infiltrated with *A. tumefaciens* containing the plasmid LIII GFP and LIII GFP+RNAi. Co-expression of the RNAi construct leads to silencing of the GFP signal. Scale bars = 50 µm.
(TIF)

Figure S8 **Nucleotide sequence alignment of destabilized GFP (dGFP) and recoded re-dGFP.** Boxes shaded in orange indicate modified nucleotides. The unchanged single letter amino acid (aa) code for each codon is shown below the alignment.
(EPS)

Table S1 **Backbones and dummies.**
(XLSX)

Table S2 **LI modules.**
(DOCX)

Table S3 **Primers.**
(DOCX)

File S1 **Sequences of Plasmids and Modules.** Zip archive of annotated maps of toolkit plasmids and modules (Genbank format).
(ZIP)

## References

[1] Khalil AS, Collins JJ (2010) Synthetic biology: applications come of age. Nat Rev Genet 11: 367–379. 20395970 10.1038/nrg2775 PMC2896386

[2] Haseloff J, Ajioka J (2009) Synthetic biology: history, challenges and prospects. Journal of The Royal Society Interface 6: S389–S391. 10.1098/rsif.2009.0176.focus PMC2843964 19493895

[3] Ahmad P, Ashraf M, Younis M, Hu X, Kumar A, et al (2012) Role of transgenic plants in agriculture and biopharming. Biotechnology Advances 30: 524–540. 21959304 10.1016/j.biotechadv.2011.09.006

[4] Jackson DA, Symons RH, Berg P (1972) Biochemical method for inserting new genetic information into DNA of simian virus 40: Circular SV40 DNA molecules containing lambda phage genes and the galactose operon of *Escherichia coli* . Proceedings of the National Academy of Sciences, USA 69: 2904–2909. 10.1073/pnas.69.10.2904 PMC389671 4342968

[5] Cohen SN, Chang ACY, Boyer HW, Helling RB (1973) Construction of biologically functional bacterial plasmids *in vitro* . Proceedings of the National Academy of Sciences, USA 70: 3240–3244. 10.1073/pnas.70.11.3240 PMC427208 4594039

[6] Marsischky G, LaBaer J (2004) Many paths to many clones: a comparative look at high-throughput cloning methods. Genome Research 14: 2020–2028. 15489321 10.1101/gr.2528804

[7] Hartley JL, Temple GF, Brasch MA (2000) DNA cloning using In vitro site-specific recombination. Genome Research 10: 1788–1795. 11076863 10.1101/gr.143000 PMC310948

[8] Liu Q, Li MZ, Leibham D, Cortez D, Elledge SJ (1998) The univector plasmid-fusion system, a method for rapid construction of recombinant DNA without restriction enzymes. Current biology 8: 1300. 9843682 10.1016/s0960-9822(07)00560-x

[9] Liu Q, Li MZ, Liu D, Elledge SJ (2000) Rapid construction of recombinant DNA by the univector plasmid-fusion system. In: Jeremy Thorner SDE, John NA, editors. Methods in Enzymology: Academic Press. 530–549. 10.1016/s0076-6879(00)28417-6 11075365

[10] Karimi M, Depicker A, Hilson P (2007) Recombinational cloning with plant gateway vectors. Plant Physiology 145: 1144–1154. 18056864 10.1104/pp.107.106989 PMC2151728

[11] Curtis MD, Grossniklaus U (2003) A Gateway cloning vector set for high-throughput functional analysis of genes in planta. Plant Physiology 133: 462–469. 14555774 10.1104/pp.103.027979 PMC523872

[12] Nakagawa T, Suzuki T, Murata S, Nakamura S, Hino T, et al (2007) Improved Gateway binary vectors: high-performance vectors for creation of fusion constructs in transgenic analysis of plants. Bioscience, Biotechnology, and Biochemistry 71: 2095–2100. 10.1271/bbb.70216 17690442

[13] Earley KW, Haag JR, Pontes O, Opper K, Juehne T, et al (2006) Gateway-compatible vectors for plant functional genomics and proteomics. Plant Journal 45: 616–629. 16441352 10.1111/j.1365-313X.2005.02617.x

[14] Karimi M, Inzé D, Depicker A (2002) GATEWAY vectors for *Agrobacterium*-mediated plant transformation. Trends in plant science 7: 193–195. 11992820 10.1016/s1360-1385(02)02251-3

[15] Li MZ, Elledge SJ (2007) Harnessing homologous recombination in vitro to generate recombinant DNA via SLIC. Nature Methods 4: 251–256. 17293868 10.1038/nmeth1010

[16] Gibson DG, Young L, Chuang R-Y, Venter JC, Hutchison CA, et al (2009) Enzymatic assembly of DNA molecules up to several hundred kilobases. Nature Methods 6: 343–345. 19363495 10.1038/nmeth.1318

[17] Quan J, Tian J (2009) Circular polymerase extension cloning of complex gene libraries and pathways. PLoS ONE 4: e6441. 19649325 10.1371/journal.pone.0006441 PMC2713398

[18] Zhang Y, Werling U, Edelmann W (2012) SLiCE: a novel bacterial cell extract-based DNA cloning method. Nucleic Acids Research 40: e55. 22241772 10.1093/nar/gkr1288 PMC3333860

[19] Engler C, Kandzia R, Marillonnet S (2008) A one pot, one step, precision cloning method with high throughput capability. PLoS ONE 3: e3647. 18985154 10.1371/journal.pone.0003647 PMC2574415

[20] Engler C, Gruetzner R, Kandzia R, Marillonnet S (2009) Golden gate shuffling: A one-pot DNA shuffling method based on type IIs restriction enzymes. PLoS ONE 4: e5553. 19436741 10.1371/journal.pone.0005553 PMC2677662

[21] Engler C, Marillonnet S (2011) Generation of Families of Construct Variants Using Golden Gate Shuffling. In: Lu C, Browse J, Wallis JG, editors. cDNA Libraries: Humana Press. 167–181. 10.1007/978-1-61779-065-2_11 21365490

[22] Weber E, Engler C, Gruetzner R, Werner S, Marillonnet S (2011) A Modular Cloning System for Standardized Assembly of Multigene Constructs. PLoS ONE 6: e16765. 21364738 10.1371/journal.pone.0016765 PMC3041749

[23] Sarrion-Perdigones A, Falconi EE, Zandalinas SI, Juárez P, Fernández-del-Carmen A, et al (2011) GoldenBraid: An Iterative Cloning System for Standardized Assembly of Reusable Genetic Modules. PLoS ONE 6: e21622. 21750718 10.1371/journal.pone.0021622 PMC3131274

[24] Sarrion-Perdigones A, Vazquez-Vilar M, Palací J, Castelijns B, Forment J, et al (2013) GoldenBraid 2.0: A Comprehensive DNA Assembly Framework for Plant Synthetic Biology. Plant Physiology 162: 1618–1631. 23669743 10.1104/pp.113.217661 PMC3707536

[25] Weber E, Gruetzner R, Werner S, Engler C, Marillonnet S (2011) Assembly of Designer TAL Effectors by Golden Gate Cloning. PLoS ONE 6: e19722. 21625552 10.1371/journal.pone.0019722 PMC3098256

[26] Cermak T, Doyle EL, Christian M, Wang L, Zhang Y, et al (2011) Efficient design and assembly of custom TALEN and other TAL effector-based constructs for DNA targeting. Nucleic Acids Research 39: e82. 21493687 10.1093/nar/gkr218 PMC3130291

[27] Sakuma T, Hosoi S, Woltjen K, Suzuki K-i, Kashiwagi K, et al (2013) Efficient TALEN construction and evaluation methods for human cell and animal applications. Genes to Cells 18: 315–326. 23388034 10.1111/gtc.12037

[28] Morbitzer R, Elsaesser J, Hausner J, Lahaye T (2011) Assembly of custom TALE-type DNA binding domains by modular cloning. Nucleic Acids Research 39: 5790–5799. 21421566 10.1093/nar/gkr151 PMC3141260

[29] Bernard P, Couturier M (1992) Cell killing by the F plasmid CcdB protein involves poisoning of DNA-topoisomerase II complexes. Journal of Molecular Biology 226: 735–745. 1324324 10.1016/0022-2836(92)90629-x

[30] Lütcke HA, Chow KC, Mickel FS, Moss KA, Kern HF, et al (1987) Selection of AUG initiation codons differs in plants and animals. EMBO Journal 6: 43–48. 3556162 10.1002/j.1460-2075.1987.tb04716.x PMC553354

[31] Gan S, Xie M (2009) Genetic insulator for preventing influence by another gene promoter. United States: University of Kentucky Research Foundation (Lexington, KY, US).

[32] Smith NA, Singh SP, Wang M-B, Stoutjesdijk PA, Green AG, et al (2000) Gene expression: Total silencing by intron-spliced hairpin RNAs. Nature 407: 319–320. 11014180 10.1038/35030305

[33] Maekawa T, Kusakabe M, Shimoda Y, Sato S, Tabata S, et al (2008) Polyubiquitin promoter-based binary vectors for overexpression and gene silencing in *Lotus japonicus* . Molecular Plant-Microbe Interactions 21: 375–382. 18321183 10.1094/MPMI-21-4-0375

[34] Sarah MR, Paul WN, Robert MY, Jef DB, Joel SB (2010) GeneDesign 3.0 is an updated synthetic biology toolkit. Nucleic Acids Research 38: 2603–2606. 20211837 10.1093/nar/gkq143 PMC2860129

[35] Grote A, Hiller K, Scheer M, Münch R, Nörtemann B, et al (2005) JCat: a novel tool to adapt codon usage of a target gene to its potential expression host. Nucleic Acids Research 33: W526–W531. 15980527 10.1093/nar/gki376 PMC1160137

[36] Voinnet O, Rivas S, Mestre P, Baulcombe D (2003) An enhanced transient expression system in plants based on suppression of gene silencing by the p19 protein of tomato bushy stunt virus. Plant Journal 33: 949–956. 12609035 10.1046/j.1365-313x.2003.01676.x

[37] Lakatos L, Szittya G, Silhavy D, Burgyan J (2004) Molecular mechanism of RNA silencing suppression mediated by p19 protein of tombusviruses. EMBO J 23: 876–884. 14976549 10.1038/sj.emboj.7600096 PMC381004

[38] Yan P, Shen W, Gao X, Li X, Zhou P, et al (2012) High-throughput construction of intron-containing hairpin RNA vectors for RNAi in plants. PLoS ONE 7: e38186. 22675447 10.1371/journal.pone.0038186 PMC3364983

[39] Jackson AL, Bartz SR, Schelter J, Kobayashi SV, Burchard J, et al (2003) Expression profiling reveals off-target gene regulation by RNAi. Nat Biotech 21: 635–637. 10.1038/nbt831 12754523

[40] Ma Y, Creanga A, Lum L, Beachy PA (2006) Prevalence of off-target effects in Drosophila RNA interference screens. Nature 443: 359–363. 16964239 10.1038/nature05179

[41] Editorial (2003) Whither RNAi? Nature Cell Biology 5: 489–490. 12776118 10.1038/ncb0603-490

[42] Lampropoulos A, Sutikovic Z, Wenzl C, Maegele I, Lohmann JU, et al (2013) GreenGate - A novel, versatile, and efficient cloning system for plant transgenesis. PLoS ONE 8: e83043. 24376629 10.1371/journal.pone.0083043 PMC3869738

[43] Karimi M, Inzé D, Depicker A (2002) GATEWAY” vectors for *Agrobacterium*-mediated plant transformation. Trends in plant science 7: 193–195. 11992820 10.1016/s1360-1385(02)02251-3

[44] Gerard RL, Pascal AS, Robert AL (1991) A DNA Transformation–Competent *Arabidopsis* Genomic Library in *Agrobacterium* . Nature Biotechnology 9: 963–967. 10.1038/nbt1091-963 1368724

[45] Stougaard J, Abildsten D, Marcker K (1987) The *Agrobacterium rhizogenes* pRi TL-DNA segment as a gene vector system for transformation of plants. Molecular and General Genetics MGG 207: 251–255.

[46] Díaz CL, Grønlund M, Schulaman HRM, Spaink HP (2005) Induction of hairy roots for symbiotic gene expression studies. *Lotus japonicus* Handbook. Dordrecht, The Netherlands: Springer-Verlag. 261–277.

[47] Clough SJ, Bent AF (1998) Floral dip: a simplified method for *Agrobacterium*-mediated transformation of *Arabidopsis thaliana* . Plant Journal 16: 735–743. 10069079 10.1046/j.1365-313x.1998.00343.x

[48] Murashige T, Skoog F (1962) A Revised Medium for Rapid Growth and Bio Assays with Tobacco Tissue Cultures. Physiologia Plantarum 15: 473–497.

