# Supplementary figures and images for: A Modular Plasmid Assembly Kit for Multigene Expression, Gene Silencing and Silencing Rescue in Plants
Source: PLoS One. 2014 Feb 13;9(2):e88218. doi: 10.1371/journal.pone.0088218 (PMC3923767; doi:10.1371/journal.pone.0088218)

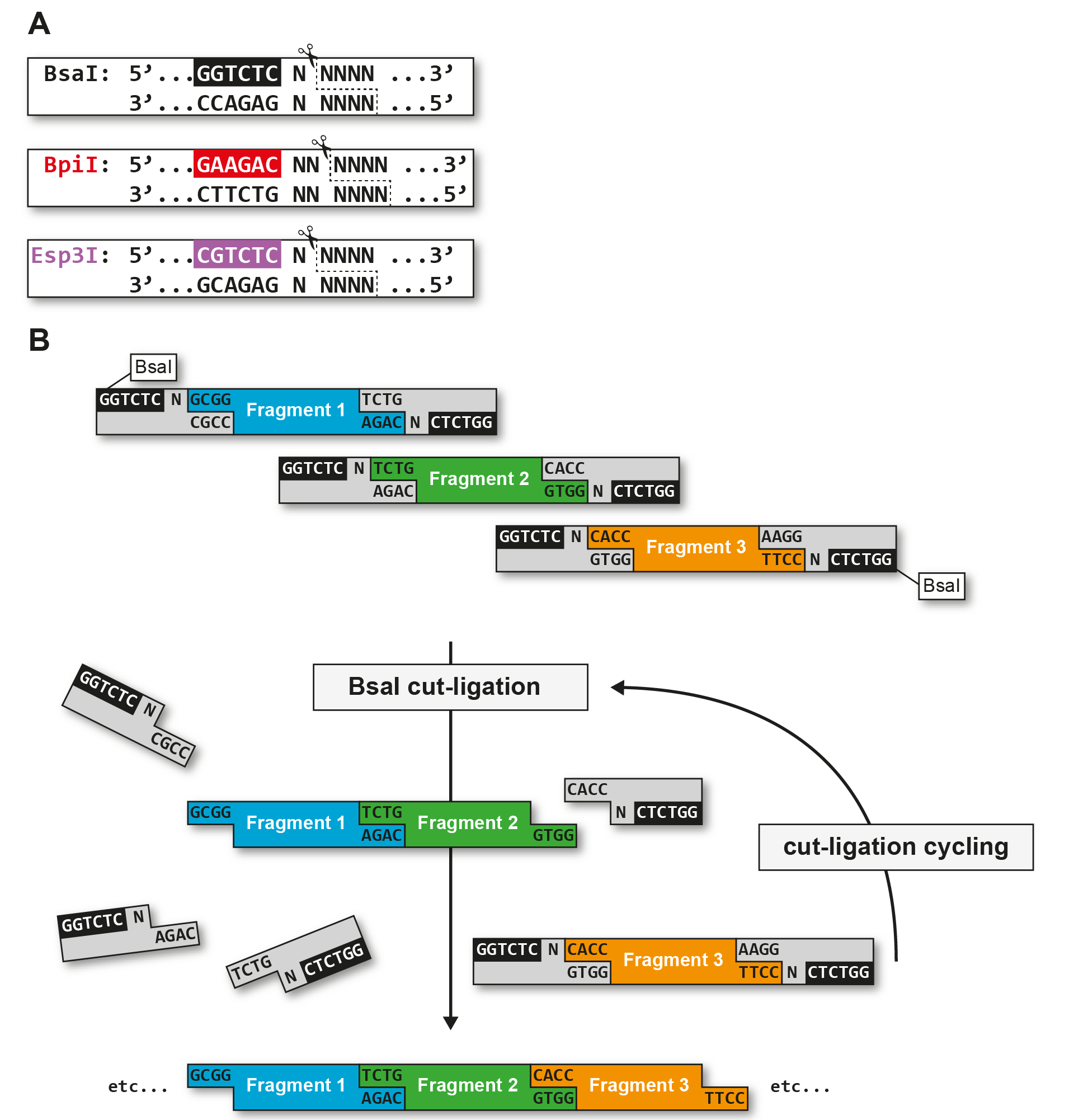

Supplement: Figure S1 — Type IIS restriction enzymes and Golden Gate cut-ligation. A) Recognition and restriction sites of type IIS endonucleases BsaI, BpiI and Esp3I. B) Cut-ligations combine restriction and ligation in one single cyclical reaction, allowing for efficient assembly of multiple fragments and continuous cleavage of undesired starting products. (TIF) [file pone.0088218.s001.tif]

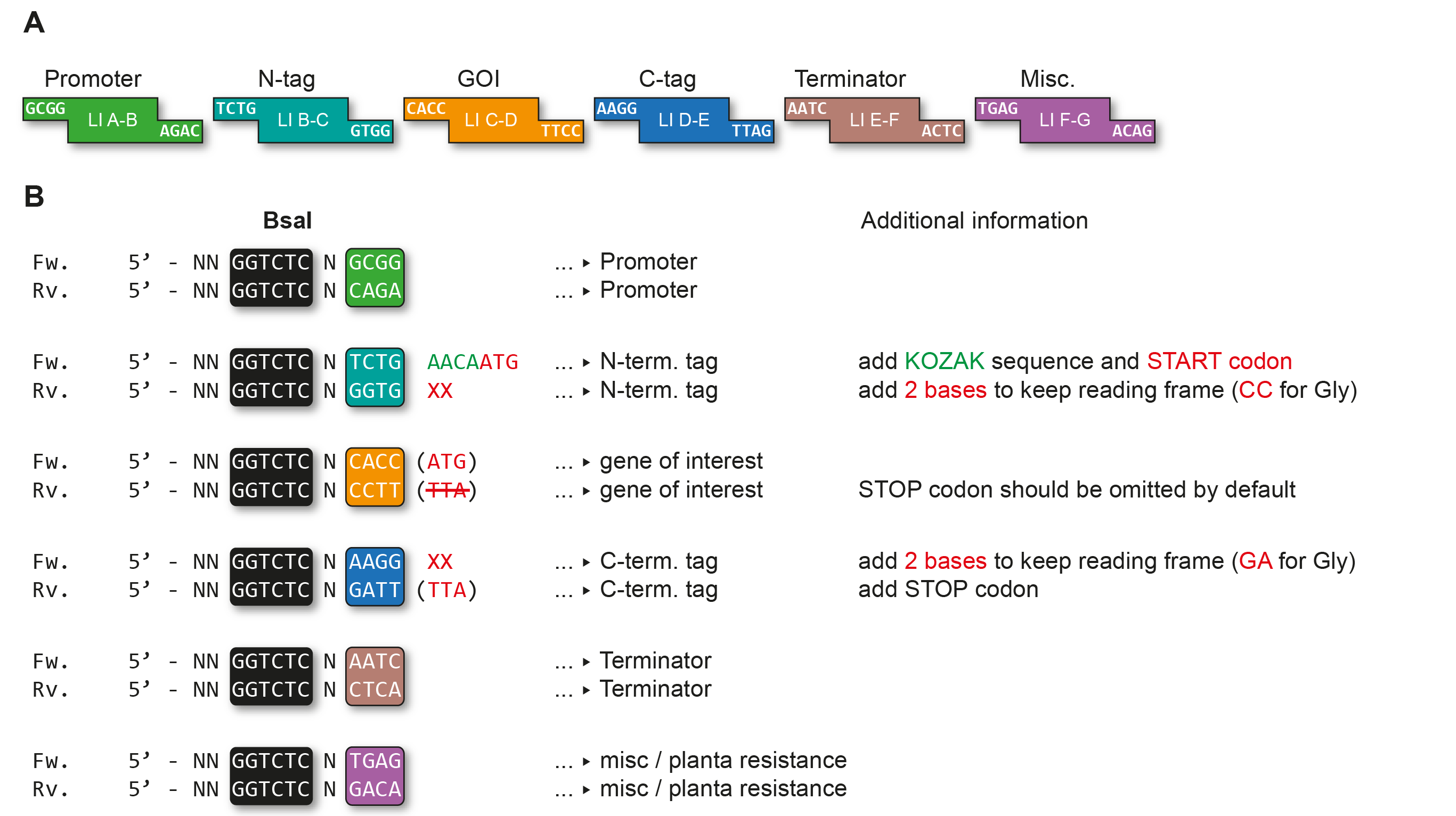

Supplement: Figure S2 — LI modules and primer design for amplification of template sequences without mutagenesis. A) Classes of LI modules with their respective overhangs after BsaI cleavage. B) Outer sequence of the forward (Fw.) and reverse primers (Rv.) used to amplify a particular LI module without mutagenesis. As blunt end subcloning sometimes resulted in the loss of the terminal 5′ bases, 2 additional bases (NN) were included at the 5′ position of the primers, in order to increase the number of correct clones. (TIF) [file pone.0088218.s002.tif]

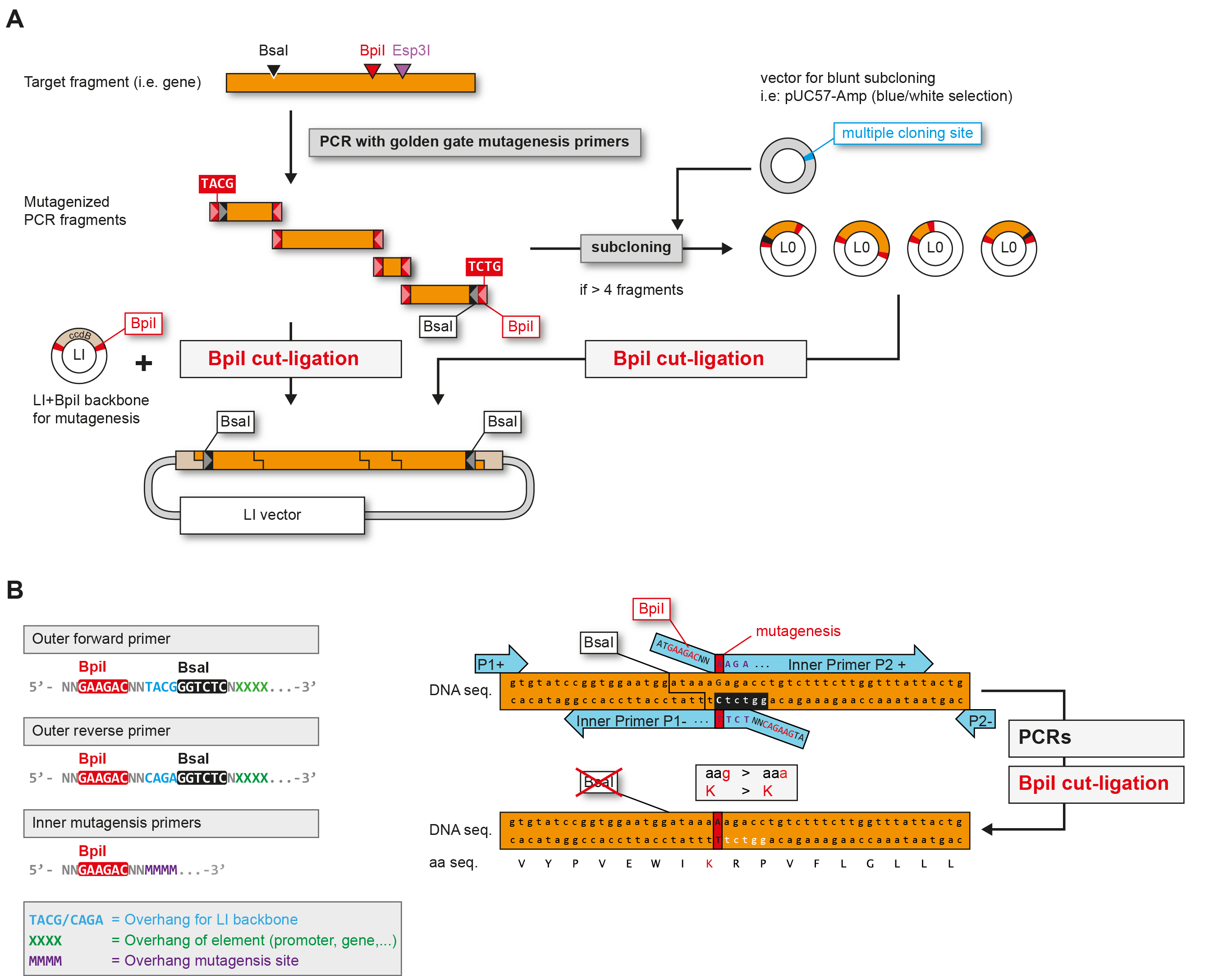

Supplement: Figure S3 — Generation of LI modules with removal of type IIS sites. A) A target sequence with undesired restriction sites is amplified in separate PCR reactions with mutagenic primers containing BpiI sites. Assembly of the mutagenized fragments is done by BpiI cut ligation into the LI+BpiI vector backbone, either directly from PCR fragments or after blunt-end subcloning into a L0 vector. B) Details on primer design and mutagenesis. The outer primers contain an additional BsaI site, which is required to assemble the finished LI module into a LII vector. The inner primers introduce a silent mutation into the target sequence in order to remove the original type IIS restriction site. For details on the specific LI element overhangs as well as additional consideration for primer design see Figure S2. (TIF) [file pone.0088218.s003.tif]

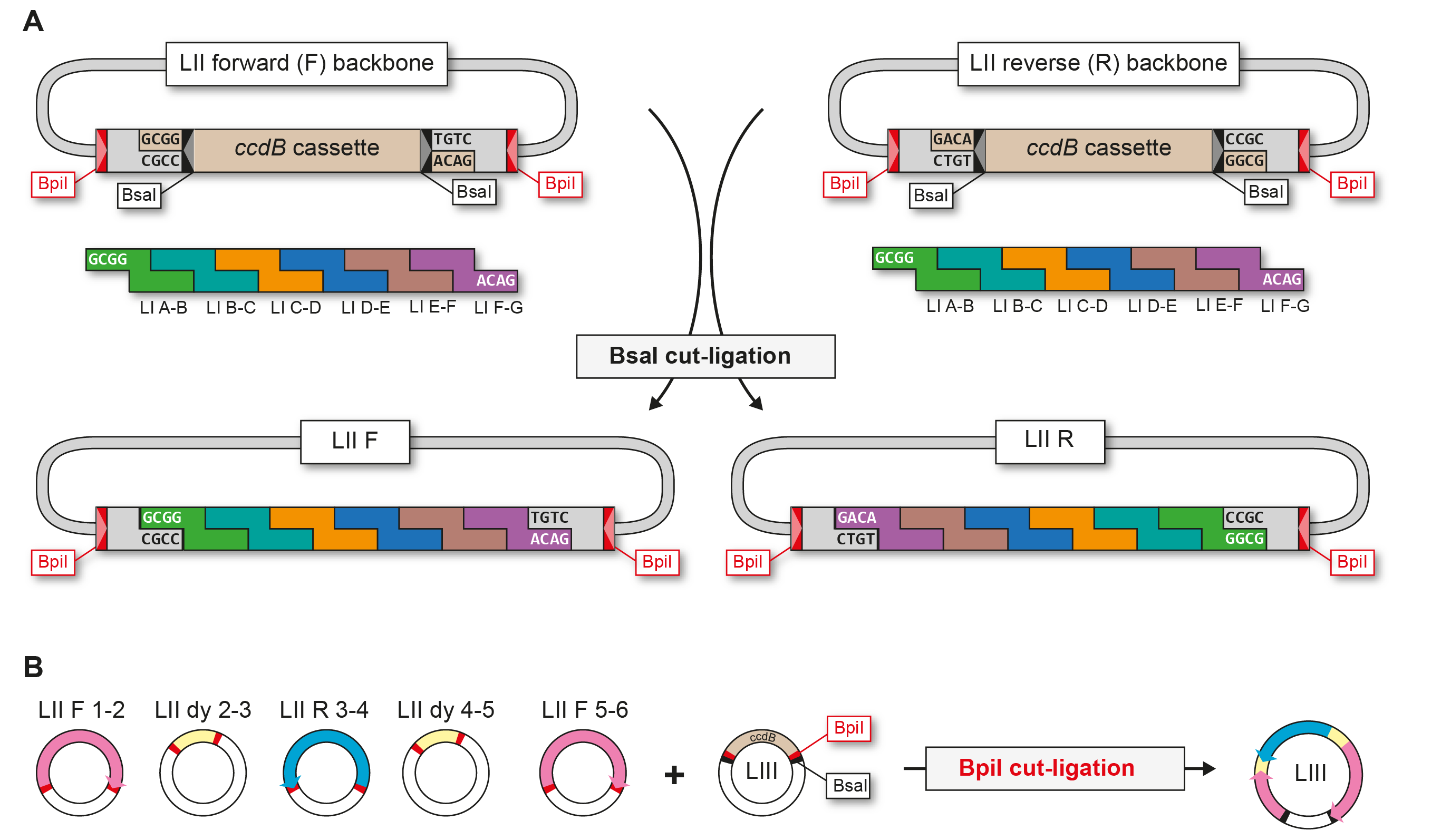

Supplement: Figure S4 — LII vector backbones define the orientation of the transcription units in LIII constructs. A) LII backbones vectors are available in a forward (F) and reverse (R) version. In the LII R backbone BsaI overhangs are flipped, thus resulting in the insertion of the LI modules in inverse orientation after BsaI cut-ligation. B) Exemplary construction of LII assemblies in different orientations into a LIII vector backbone. (TIF) [file pone.0088218.s004.tif]

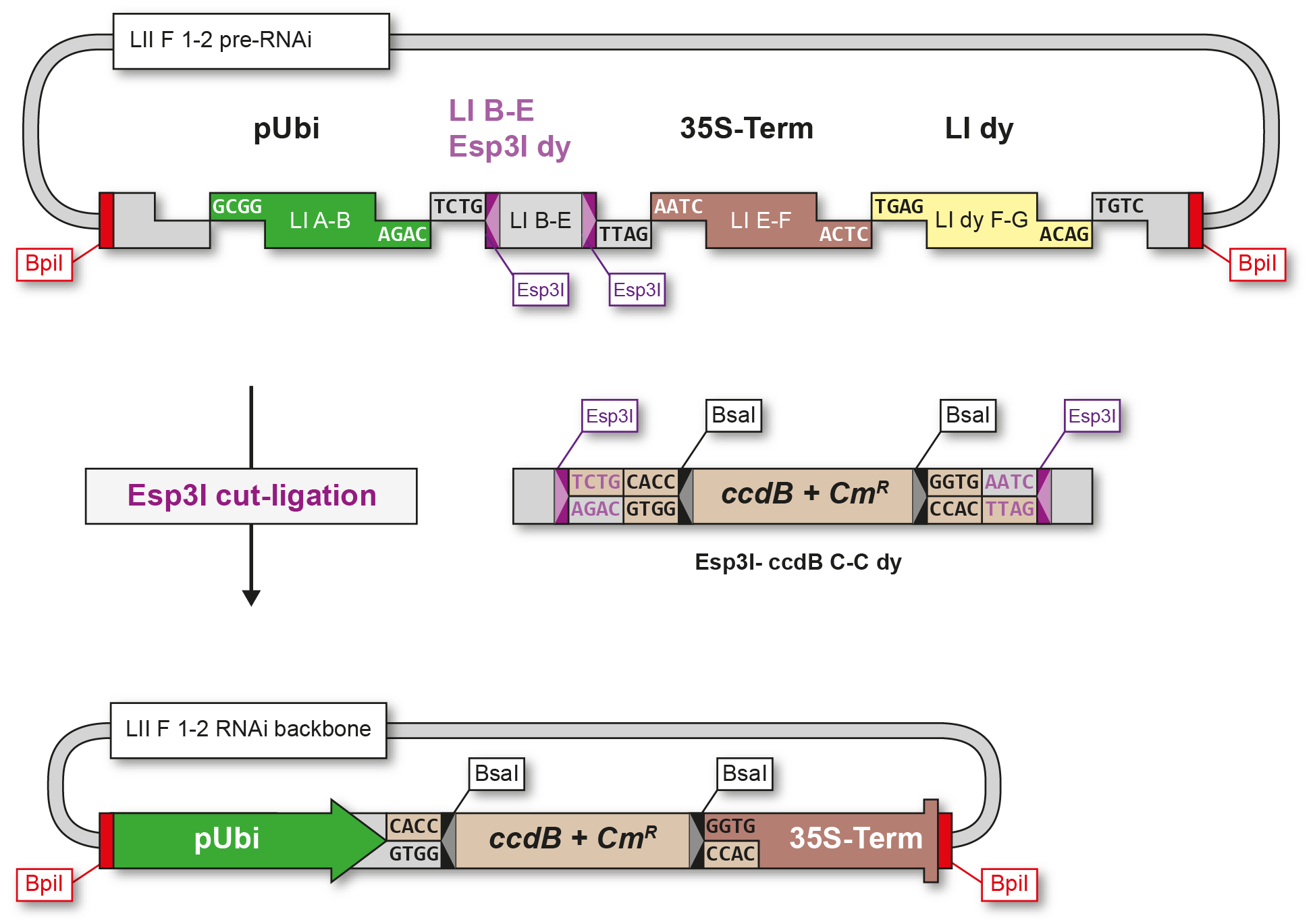

Supplement: Figure S5 — Vector backbone construction for RNAi mediated gene silencing. First a preliminary construct (LII F 1–2 RNAi-pre) was assembled out of a LII F 1–2 backbone, a ubiquitin promoter (pUbi) module (LI A-B), a 35S-terminator (LI E-F), a LI dy F-G and a custom dummy (LI B-E Esp3I dy) module by BsaI cut-ligation. The LI B-E Esp3I dy contains BsaI sites which fit to the B and E overhangs of LI modules as well as inner Esp3I sites which generate the same B and E overhangs when cleaved. In a second step a ccdB cassette containing matching Esp3I and additional inner BsaI sites was combined with the LII F 1–2 RNAi-pre vector by Esp3I cut-ligation. The finished LII F 1–2 RNAi vector contains the ccdB cassette flanked by BsaI sites with the fusion sites CACC and GGTG, which can be used to insert 2 copies of a LI C-D element together with an intron element. (TIF) [file pone.0088218.s005.tif]

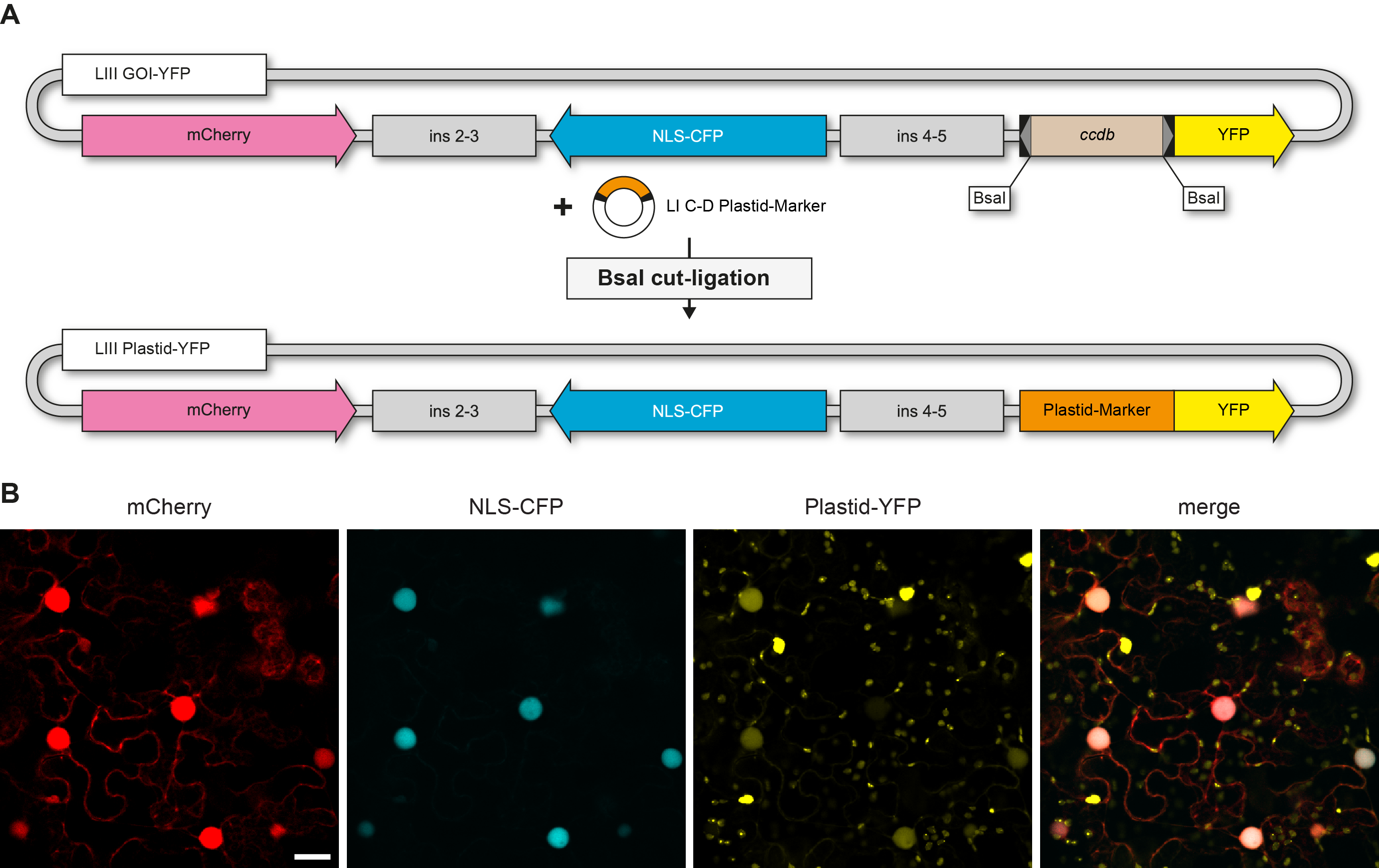

Supplement: Figure S6 — Expression of mCherry, CFP and YFP using a custom LIII binary vector. A) The LIII Plastid-YFP binary plasmid was constructed by insertion of a LI C-D Plastid-Marker (genomic sequence of plastid localized protein from L. japonicus) module via BsaI cut-ligation into the LIII-GOI-YFP vector backbone, which was preassembled with free mCherry and NLS-2xCFP fluorescence markers B) CLSM images of N. benthamiana transformed with the LIII Plastid-YFP construct 2 days after Agrobacterium mediated transformation. Scale bar = 25 µm. (TIF) [file pone.0088218.s006.tif]

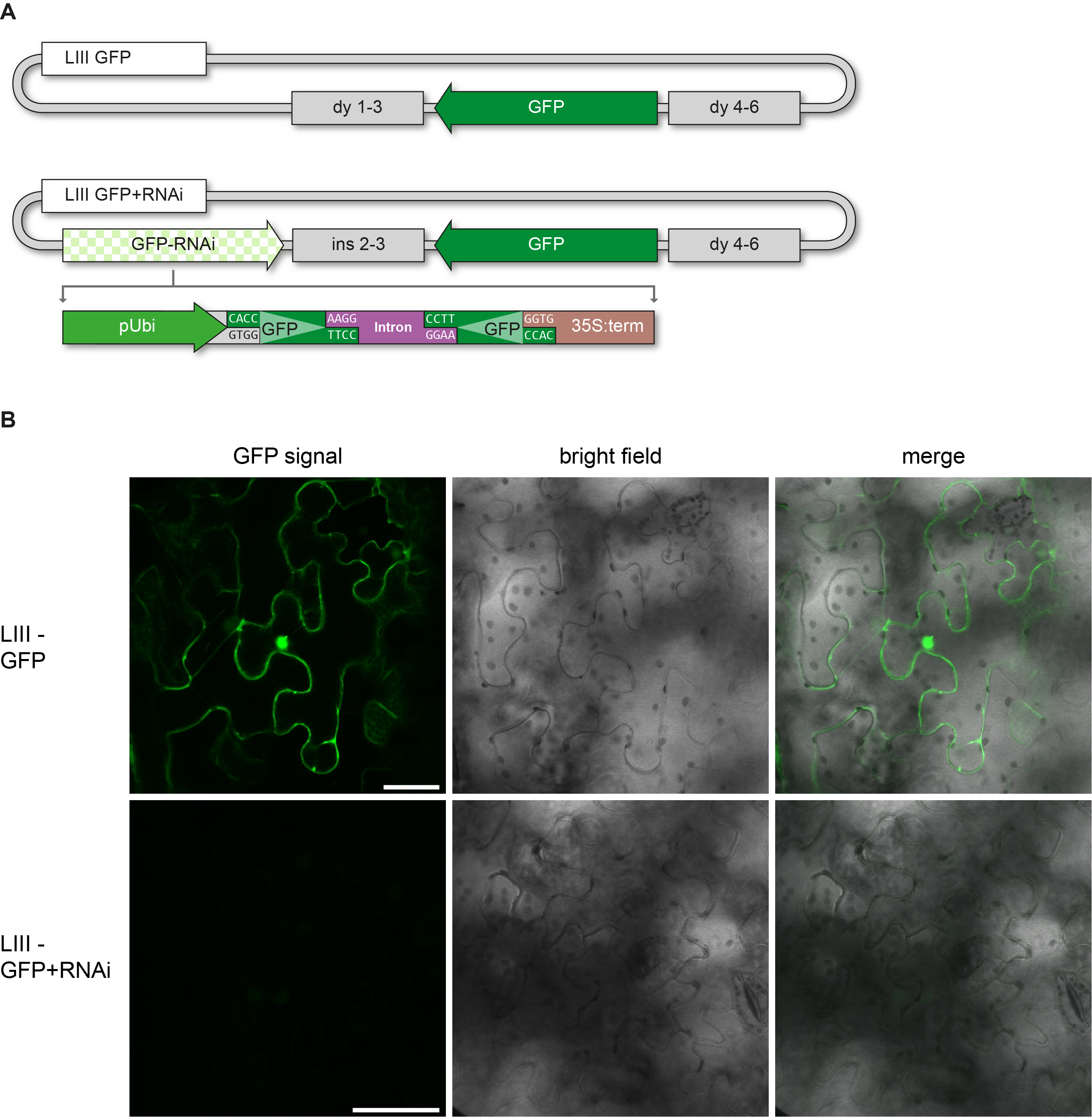

Supplement: Figure S7 — RNAi mediated knock-down of GFP. A) Schematic representation of LIII binary constructs LIII GFP and LIII GFP+RNAi. B) CLSM images of N. benthamiana leaves infiltrated with A. tumefaciens containing the plasmid LIII GFP and LIII GFP+RNAi. Co-expression of the RNAi construct leads to silencing of the GFP signal. Scale bars = 50 µm. (TIF) [file pone.0088218.s007.tif]
