# Supplementary material for: A Modular Plasmid Assembly Kit for Multigene Expression, Gene Silencing and Silencing Rescue in Plants
Source: PLoS One. 2014 Feb 13;9(2):e88218. doi: 10.1371/journal.pone.0088218 (PMC3923767; doi:10.1371/journal.pone.0088218)
Supplement: Table S2 — LI modules. (DOCX) [file pone.0088218.s010.docx]

**Supplementary Table: 2: LI modules**

| **Label** | **Name** | **Origin / Comment** |
| --- | --- | --- |
| **Promoters** |  |  |
| G007 | LI A-B pUbi | *L. japonicus* polyubiquitin promoter [[1](#_ENREF_1)] |
| G005 | LI A-B p35S | cauliflower mosaic virus 35S promoter [[2](#_ENREF_2)] |
| G009 | LI A-C p35S | cauliflower mosaic virus 35S promoter [[2](#_ENREF_2)] |
| G080 | LI A-B pNOS | nopaline synthase promoter [[3](#_ENREF_3)] |
| G052 | LI A-B pEF1 | promoter region of *A. thaliana EF1*α1 /AT1G07940.1; this work |
|  |  |  |
| **Fluorescent tags and markers** | |  |
| G028 | LI B-C GFP | sGFP + 16 aa GS linker [[4](#_ENREF_4)] |
| G029 | LI B-C YFP | YFP-Venus + 16 aa GS linker [[5](#_ENREF_5)] |
| G030 | LI B-C T-Saphire | T-saphhire + 16 aa GS linker [[6](#_ENREF_6)] |
| G031 | LI B-C Cerulean | CFP-Cerulean + 16 aa GS linker [[7](#_ENREF_7)] |
| G032 | LI B-C mCherry | mCherry + 16 aa GS linker [[8](#_ENREF_8)] |
| G033 | LI B-C mOrange | mOrange + 16 aa GS linker [[8](#_ENREF_8)] |
| G019 | LI C-D GFP | sGFP [[4](#_ENREF_4)] |
| G020 | LI C-D YFP | YFP Venus [[5](#_ENREF_5)] |
| G021 | LI C-D T-Sapphire | T-saphhire [[6](#_ENREF_6)] |
| G022 | LI C-D Cerulean | CFP Cerulean [[7](#_ENREF_7)] |
| G023 | LI C-D mCherry | mCherry [[8](#_ENREF_8)] |
| G024 | LI C-D mOrange | mOrange [[8](#_ENREF_8)] |
|  |  |  |
| G053 | LI C-D GFP noATG | sGFP without Start codon [[4](#_ENREF_4)] |
| G054 | LI C-D YFP noATG | YFP Venus without Start codon [[5](#_ENREF_5)] |
| G055 | LI C-D T-Sapphire noATG | T-saphhire without Start codon [[6](#_ENREF_6)] |
| G056 | LI C-D Cerulean noATG | CFP Cerulean without Start codon [[7](#_ENREF_7)] |
| G057 | LI C-D mCherry noATG | mCherry without Start codon [[8](#_ENREF_8)] |
| G058 | LI C-D mOrange noATG | mOrange without Start codon [[8](#_ENREF_8)] |
| G078 | LI C-D dGFP | destablized GFP [[9](#_ENREF_9)] |
| G096 | LI C-D re dGFP | destablized GFP (recoded); this work |
|  |  |  |
| G011 | LI D-E GFP | 16 aa GS linker + GFP [[4](#_ENREF_4)] |
| G012 | LI D-E YFP | 16 aa GS linker + YFP Venus [[5](#_ENREF_5)] |
| G013 | LI D-E T-Sapphire | 16 aa GS linker + T-Sapphire [[6](#_ENREF_6)] |
| G014 | LI D-E Cerulean | 16 aa GS linker + CFP Cerulean [[7](#_ENREF_7)] |
| G025 | LI D-E mCherry | 16 aa GS linker + mCherry [[8](#_ENREF_8)] |
| G026 | LI D-E mOrange | 16 aa GS linker + mOrange [[8](#_ENREF_8)] |
|  |  |  |
| G090 | LI C-D 1-10GFP | superfolder GFP 1-10 domain [[10](#_ENREF_10)] |
| G091 | LI C-D 1-10GFP noATG | superfolder GFP 1-10 domain without start codon [[10](#_ENREF_10)] |
| G076 | LI D-E 1-10GFP | 16 aa GS linker + superfolder GFP 1-10 domain [[10](#_ENREF_10)] |
| G042 | LI C-D 11GFP | superfolder GFP 11 domain [[10](#_ENREF_10)] |
| G041 | LI D-E 11GFP | FLAG tag + and short linker + superfolder GFP 11 domain [[10](#_ENREF_10)] |
|  |  |  |
| **Other tags** |  |  |
| G065 | LI B-C 6xHIS | polyhistidine (6x) tag [[11](#_ENREF_11)] |
| G066 | LI D-E 6xHIS | polyhistidine (6x) tag [[11](#_ENREF_11)] |
| G067 | LI B-C HA | human influenza hemagglutinin (HA) tag (YPYDVPDYA) |
| G068 | LI D-E HA | human influenza hemagglutinin (HA) tag (YPYDVPDYA) |
| G069 | LI B-C c-MYC | c-MYC epitope tag (EQKLISEEDL) |
| G070 | LI D-E c-MYC | c-MYC epitope tag (EQKLISEEDL) |
| G060 | LI B-C N-NLS | Nuclear localization signal (NLS) of SV40 large T antigen [[12](#_ENREF_12)] |
| G062 | LI B-C N-NES (I) | Nuclear Export Signal (NES) of heat stable inhibitor (PKI) [[13](#_ENREF_13)] |
| G061 | LI B-C N-NES (II) | CPK17-N-terminal + NES for enhanced export [[14](#_ENREF_14)] |
|  |  |  |
| G035 | LI D-E C-NLS | NLS of SV40 large T antigen [[12](#_ENREF_12)] |
| G038 | LI D-E C-NES | NES of heat stable inhibitor (PKI) [[13](#_ENREF_13)] |
|  |  |  |
| **Terminators** |  |  |
| G006 | LI E-F nos-T | Nopaline synthase terminator [[3](#_ENREF_3)] |
| G045 | LI E-F HSP-T | Heat Shock Protein terminator *A.thaliana* [[15](#_ENREF_15)] |
| G059 | LI E-F 35S-T | Terminator of Cauliflower mosaic virus CaMV [[2](#_ENREF_2)] |
|  |  |  |
| **Misc** |  |  |
| G079 | LI B-E GUSi | β-glucuronidase (GUS) with intron [[16](#_ENREF_16)] |
| G050 | LI Intron | Intron elemet for construction of silencing vectors (intron1 of *AtWRKY33)[*[*1*](#_ENREF_1)*]* |
|  |  |  |
| **In planta resistances** | |  |
| G003 | LI F-G Neo | neomycin phosphotransferase II (nptII) [[17](#_ENREF_17)] |
| G095 | LI F-G Hygro | hygromycin phosphotranferase (hptII) (pCAMBIA) |

1. Maekawa T, Kusakabe M, Shimoda Y, Sato S, Tabata S, et al. (2008) Polyubiquitin promoter-based binary vectors for overexpression and gene silencing in *Lotus japonicus*. Molecular Plant-Microbe Interactions 21: 375-382.

2. Guilley H, Dudley RK, Jonard G, Balàzs E, Richards KE (1982) Transcription of cauliflower mosaic virus DNA: detection of promoter sequences, and characterization of transcripts. Cell 30: 763-773.

3. Depicker A, Stachel S, Dhaese P, Zambryski P, Goodman HM (1982) Nopaline synthase: transcript mapping and DNA sequence. Journal of molecular and applied genetics 1: 561-573.

4. Chiu W-l, Niwa Y, Zeng W, Hirano T, Kobayashi H, et al. (1996) Engineered GFP as a vital reporter in plants. Current Biology 6: 325-330.

5. Nagai T, Ibata K, Park ES, Kubota M, Mikoshiba K, et al. (2002) A variant of yellow fluorescent protein with fast and efficient maturation for cell-biological applications. Nature Biotechnology 20: 87-90.

6. Zapata-Hommer O, Griesbeck O (2003) Efficiently folding and circularly permuted variants of the Sapphire mutant of GFP. BMC Biotechnology 3: 5.

7. Rizzo MA, Springer GH, Granada B, Piston DW (2004) An improved cyan fluorescent protein variant useful for FRET. Nature Biotechnology 22: 445-449.

8. Shaner NC, Campbell RE, Steinbach PA, Giepmans BNG, Palmer AE, et al. (2004) Improved monomeric red, orange and yellow fluorescent proteins derived from *Discosoma* sp. red fluorescent protein. Nature Biotechnology 22: 1567-1572.

9. Mussolino C, Morbitzer R, Lütge F, Dannemann N, Lahaye T, et al. (2011) A novel TALE nuclease scaffold enables high genome editing activity in combination with low toxicity. Nucleic Acids Research 39: 9283-9293.

10. Cabantous S, Terwilliger TC, Waldo GS (2005) Protein tagging and detection with engineered self-assembling fragments of green fluorescent protein. Nature Biotechnology 23: 102-107.

11. Hochuli E, Bannwarth W, Dobeli H, Gentz R, Stuber D (1988) Genetic approach to facilitate purification of recombinant proteins with a novel metal chelate adsorbent. Nature Biotechnology 6: 1321-1325.

12. Kalderon D, Roberts BL, Richardson WD, Smith AE (1984) A short amino acid sequence able to specify nuclear location. Cell 39: 499-509.

13. Wen W, Meinkotht JL, Tsien RY, Taylor SS (1995) Identification of a signal for rapid export of proteins from the nucleus. Cell 82: 463-473.

14. Mehlmer N, Parvin N, Hurst CH, Knight MR, Teige M, et al. (2012) A toolset of aequorin expression vectors for in planta studies of subcellular calcium concentrations in *Arabidopsis thaliana*. Journal of Experimental Botany 63: 1751-1761.

15. Nagaya S, Kawamura K, Shinmyo A, Kato K (2010) The *HSP* terminator of *Arabidopsis thaliana* increases gene expression in plant cells. Plant and Cell Physiology 51: 328-332.

16. Ohta S, Mita S, Hattori T, Nakamura K (1990) Construction and expression in tobacco of a β-glucuronidase (GUS) reporter gene containing an intron within the coding sequence. Plant and Cell Physiology 31: 805-813.

17. Chen P-Y, Wang C-K, Soong S-C, To K-Y (2003) Complete sequence of the binary vector pBI121 and its application in cloning T-DNA insertion from transgenic plants. Molecular Breeding 11: 287-293.
