# Supplementary material for: A Modular Plasmid Assembly Kit for Multigene Expression, Gene Silencing and Silencing Rescue in Plants
Source: PLoS One. 2014 Feb 13;9(2):e88218. doi: 10.1371/journal.pone.0088218 (PMC3923767; doi:10.1371/journal.pone.0088218)
Supplement: Table S3 — Primers. (DOCX) [file pone.0088218.s011.docx]

Supplementary Table 3: Primers and templates for GG toolkit generation

| **Construct** | **Primer** | **Primer Sequence 5'- 3'** | **Template** | **Comment** |
| --- | --- | --- | --- | --- |
|  |  |  |  |  |
| **LI backbone and dummies** |  |  |  |  |
| pUC57 (Gent) + Esp3I | GentR BspHI + | GGTCATGAGACGCACACCGTGGAAACGG | pBBR-5-MCS [[1](#_ENREF_1)] | replaced *bla* resistance in pUC57 |
|  | GentR BspHI - | CCTCATGAGCGGCGTTGTGACAATTTACCG | pBBR-5-MCS [[1](#_ENREF_1)] |  |
| pUC57 (Gent) | Gentmut + | GGTCACAGCTTGTCTGTAAGC | pUC57 (Gent) + Esp3I | Esp3I site removal |
|  | Gentmut - | TTCTCCGGGAGCTGCAT | pUC57 (Gent) + Esp3I |  |
| pENTR-BsaI | GW BsaI CACC + | CACCTGAGACCTGCGGCCCCTCGAGGG | pENTR-D/TOPO (Invitrogen) | Introductio of BsaI sites and internal BsaI site removal |
|  | GW BsaI AAGG - | TGAGACCAGGTCGACCTGCAGAC | pENTR-D/TOPO (Invitrogen) |  |
|  | BsaI-del + | CAGTGTGCCGGTATCCGTTATCGGGGAAG | pENTR-D/TOPO (Invitrogen) |  |
|  | BsaI-del - | CTTCCCCGATAACGGATACCGGCACACTG | pENTR-D/TOPO (Invitrogen) |  |
| LI+Bpi | pUC+BpiI fw | TACGAAGTCTTCACAAGTTTGTACAAAAAAGCTGAACG | pENTR-BsaI | ligation into pUC57 (Gent) |
|  | pUC+BpiI rv | CAGAATGTCTTCCCAACCACTTTGTACAAGAAAGCTGAACG | pENTR-BsaI | ligation into pUC57 (Gent) |
| LI dy A-B | LI A-B dy + | ATGGTCTCAGCGGAACATCTGAGAGACCTGCAAGCTTGGCGTAATCAT | pUC57 (Gent) | amplified with LI dy - |
| LI dy B-C | LI B-C dy + | TTTGGTCTCTTCTGGAAGCACCTGAGACCGGGCCCGTCGACTGCAGAGG | pUC57 (Gent) | amplified with LI dy - |
| LI dy C-D | LI C-D dy + | TTTGGTCTCTCACCTGACCTAAGCTAGCCTAGAAGGTGAGACCGGGCCCGTCGACTGCAGAGG | pUC57 (Gent) | amplified with LI dy - |
| LI dy D-E | LI D-E dy + | TAGGTCTCTAAGGGCTAAGACCTAAGCTAGCCTAGAATCTGAGACCTGCAAGCTTGGCGTAATCAT | pUC57 (Gent) | amplified with LI dy - |
| LI dy F-G | LI F-G dy + | TTTGGTCTCTTGAGTGACCTAAGCTAGCCTAGTGTCTGAGACCGGGCCCGTCGACTGCAGAGG | pUC57 (Gent) | amplified with LI dy - |
|  | LI dy - | GGGATCCGATATCTAGATGC | pUC57 (Gent) |  |
|  |  |  |  |  |
| **Expression Vector Backbones** |  |  |  |  |
| Stage 1: LIIIa and LIIIb (both obsolete) vector backbones, were assembled as shown below | pUB 1 + | TTTGGTCTC TGATAGAAGACTTTCTGTTTAAACTATCAGTGTTTGACAGGATATATTGG | pUB-GW-HYG [[2](#_ENREF_2)] |  |
|  | pUB 1 - | AAAGGTCTCGAAGGCTGCACTGAACGTCAGA | pUB-GW-HYG [[2](#_ENREF_2)] |  |
|  | pUB 2 + | TTTGGTCTCCCCTTCTTCTGAAAACGACATGTC | pUB-GW-HYG [[2](#_ENREF_2)] |  |
|  | pUB 2 - | AAAGGTCTCGATGTGCGCGGGCGGC | pUB-GW-HYG [[2](#_ENREF_2)] |  |
|  | pUB 3 + | TTTGGTCTCCACATCTCAACCGTGCGGC | pUB-GW-HYG [[2](#_ENREF_2)] |  |
|  | pUB 3 - | AAAGGTCTCTCGCCAGCTCGTCGGTCAC | pUB-GW-HYG [[2](#_ENREF_2)] |  |
|  | pUB 4 + | TTTGGTCTCCGGCGAGGTGATCCGCTACG | pUB-GW-HYG [[2](#_ENREF_2)] |  |
|  | pUB 4 - | AAAGGTCTCAGAGTCCTTTTCGACCTTTTTCCCCTG | pUB-GW-HYG [[2](#_ENREF_2)] |  |
|  | pUB 5 + | TTTGGTCTCTACTCTTTCCTGTGGATAGCACGTAC | pUB-GW-HYG [[2](#_ENREF_2)] |  |
|  | pUB 5 - | AAAGGTCTCGACTGTCTCCGGGAGCTGC | pUB-GW-HYG [[2](#_ENREF_2)] |  |
|  | pUB 6 + | TTTGGTCTCACAGTCACAGCTTGTCTGTAAGC | pUB-GW-HYG [[2](#_ENREF_2)] |  |
|  | pUB 6 - | AAAGGTCTCATACTCCATTTAAAGATCCGCGCG | pUB-GW-HYG [[2](#_ENREF_2)] |  |
|  | pUB 7 + | TTTGGTCTCGAGTATCTTCTTCCCAGTTTTCGC | pUB-GW-HYG [[2](#_ENREF_2)] |  |
|  | pUB 7 - | AAAGGTCTCTGCGCGAAGACAACGTAGTCTAAGCGTCAATTTGTTTACACCAC | pUB-GW-HYG [[2](#_ENREF_2)] |  |
|  | ccdb + | TTTGGTCTCTGCGCACAAGTTTGTACAAAAAAGCTGAA | pENTR-BsaI |  |
|  | ccdb - | AAAGGTCTCATATCAACCACTTTGTACAAGAAAGCTGA | pENTR-BsaI |  |
|  | LIII bb + | TTTGGTCTCTGATAGAAGACTTTCTGGTTTAAACTATCAGTGTTTGACAGGA | pICH50505 (ICON Genetics) |  |
|  | LIII bb - | AAAGGTCTCTGCGCGAAGACAACGTAATCCACCCCAGTACATTAAAAA | pICH50505 (ICON Genetics) |  |
| LIIIa (obsolete) BsaI cut-ligation: 1 + 2 + 3 + 4 + 5 + 6 +7 + ccdb | | |  |  |
| LIIIb (obsolete) BsaI cut-ligation: bb + ccdb | |  |  |  |
|  |  |  |  |  |
| Stage 2: Final Expression vector backbones were assembled as shown below | A fw | ATGGTCTCATGCCATTTTTGGGGTGAG | LIII b (obsolete) |  |
|  | A rv | TAGGTCTCAATCCACCCCAGTACATTAAAAA | LIII b (obsolete) |  |
|  | B + E fw | ATGGTCTCAGGATTACGTGAGACGATACGTCTCATCTGGTTTAAACTATCAGTGTTTGACAGGA | LIII b (obsolete) |  |
|  | B rv | TAGGTCTCAATGATCTAGGTGAAGATCCTTTTTGA | LIII b (obsolete) |  |
|  | C-s fw | ATGGTCTCATCATCGGAAGAACGGCAACTAAG | pK7WG2D,1 [[3](#_ENREF_3)] |  |
|  | C-s rv | TAGGTCTCAGGCAGAAGATCCTTTGATCTTTTCTACGG | pK7WG2D,1 [[3](#_ENREF_3)] |  |
|  | C-k1 fw | ATGGTCTCATCATAAAATCATTATTTGCCATCCA | pENTR-BsaI |  |
|  | C-k1 rv | TAGGTCTCAGCAAGACGAAATACGCGATC | pENTR-BsaI |  |
|  | C-k2 fw | ATGGTCTCATTGCTCAGGCGCAATCAC | pENTR-BsaI |  |
|  | C-k2 rv | TAGGTCTCAGGCAGGGGTCTGACGCTCAGTG | pENTR-BsaI |  |
|  | D fw | ATGGTCTCATGCCGGTAACATGAGCAAAGTCTG | LIII a (obsolete) |  |
|  | D rv | TAGGTCTCAATCCGTCTAAGCGTCAATTTGTTTACACC | LIII a (obsolete) |  |
|  | E rv | TAGGTCTCAATGAGCGTCAGACCCCGTAGAA | LIII a (obsolete) |  |
| Xpre-S BsaI cut-ligation: A + B + C-s | |  |  |  |
| Xpre-K BsaI cut-ligation: A + B + C-k1 + C-k2 | |  |  |  |
| Xpre2-S BsaI cut-ligation D + E + C-s | |  |  |  |
| Xpre2-K BsaI cut-ligation: D + E + C-k1 + C-k2 | |  |  |  |
|  |  |  |  |  |
| **LII vector construction** |  |  |  |  |
| ccdB F | L II F + | GCGGTGAGACCACAAGTTTGTACAAAAAAGCTGAA | pENTR-BsaI (ccdB cassette) |  |
|  | L II F - | GACATGAGACCCCAACCACTTTGTACAAGAAAGC | pENTR-BsaI (ccdB cassette) |  |
| LIIc F 1-2 | L II F 1-2 + | GAAGACAATACGGCGGTGAGACCACAAGTTTG | ccdB F | ligation into pAMP (Geneart) |
|  | L II F 1-2 - | GAAGACAAGCCCGACATGAGACCCCAACCACT | ccdB F |  |
| LIIc F 2-3 | LII F 2-3 + | GAAGACAAGGGCGCGGTGAGACCACAAGTTTG | ccdB F | ligation into pAMP (Geneart) |
|  | LII F 2-3 - | GAAGACAACAATGACATGAGACCCCAACCACT | ccdB F |  |
| LIIc F 3-4 | LII F 3-4 + | GAAGACAAATTGGCGGTGAGACCACAAGTTTG | ccdB F | ligation into pAMP (Geneart) |
|  | LII F 3-4 - | GAAGACAAGACAGACATGAGACCCCAACCACT | ccdB F |  |
| LIIc F 4-5 | LII F 4-5 + | GAAGACAATGTCGCGGTGAGACCACAAGTTTG | ccdB F | ligation into pAMP (Geneart) |
|  | LII F 4-5 - | GAAGACAACTCAGACATGAGACCCCAACCACT | ccdB F |  |
| LIIc F 5-6 | LII F 5-6 + | GAAGACAATGAGGCGGTGAGACCACAAGTTTG | ccdB F | ligation into pAMP (Geneart) |
|  | LII F 5-6 - | GAAGACAACAGAGACATGAGACCCCAACCACT | ccdB F |  |
|  |  |  |  |  |
| ccdB R | LII R + | GACATGAGACCACAAGTTTGTACAAAAAAGCTGAA | pENTR-BsaI (ccdB cassette) |  |
|  | LII R - | GCGGTGAGACCCCAACCACTTTGTACAAGAAAGC | pENTR-BsaI (ccdB cassette) |  |
| LIIc R 1-2 | LII R 1-2 + | GAAGACAATACGGACATGAGACCACAAGTTTGTACA | ccdB R | blunt ligation into pAMP (Geneart) |
|  | LII R 1-2 - | GAAGACAAGCCCGCGGTGAGACCCCAACC | ccdB R | blunt ligation into pAMP (Geneart) |
| LIIc R 3-4 | LII R 3-4 + | GAAGACAAATTGGACATGAGACCACAAGTTTGTACA | ccdB R | blunt ligation into pAMP (Geneart) |
|  | LII R 3-4 - | GAAGACAAGACAGCGGTGAGACCCCAACC | ccdB R | blunt ligation into pAMP (Geneart) |
| LIIc R 5-6 | LII R 5-6 + | GAAGACAATGAGGACATGAGACCACAAGTTTGTACA | ccdB R | blunt ligation into pAMP (Geneart) |
|  | LII R 5-6 - | GAAGACAACAGAGCGGTGAGACCCCAACC | ccdB R | blunt ligation into pAMP (Geneart) |
| LII F 1-2 | LII X 1-2 + | TTCGTCTCTTACGGAAGACAATACGGCGGTGAGACCAAAGCTGAACGAGAAACGTAAAA | LIIc F1-2 RNAi (ccdB) | Esp3I cut-ligation into Xpre-S / Xpre2-S |
|  | LII X 1-2 - | AACGTCTCACAGAGAAGACAAGCCCGACATGAGACCGTCGACCTGCAGACTGGC | LIIc F1-2 RNAi (ccdB) |  |
| LII R 1-2 | LIIx R 1-2 + | TTCGTCTCTTACGGAAGACAATACGGACATGAGACCAAAGCTGAACGAGAAACGTAAAA | LIIc F1-2 RNAi (ccdB) | Esp3I cut-ligation into Xpre-S / Xpre2-S |
|  | LIIx R 1-2 - | AACGTCTCACAGAGAAGACAAGCCCGCGGTGAGACCGTCGACCTGCAGACTGGC | LIIc F1-2 RNAi (ccdB) |  |
| LII F 2-3 | LIIx F 2-3 + | TTCGTCTCTTACGGAAGACAAGGGCGCGGTGAGACCAAAGCTGAACGAGAAACGTAAAA | LIIc F1-2 RNAi (ccdB) | Esp3I cut-ligation into Xpre-S / Xpre2-S |
|  | LIIx F 2-3 - | AACGTCTCACAGAGAAGACAACAATGACATGAGACCGTCGACCTGCAGACTGGC | LIIc F1-2 RNAi (ccdB) |  |
|  |  |  |  |  |
| LII R 2-3 | LIIx R 2-3+ | TTCGTCTCTTACGGAAGACAAGGGCGACATGAGACCAAAGCTGAACGAGAAACGTAAAA | LIIc F1-2 RNAi (ccdB) | Esp3I cut-ligation into Xpre-S / Xpre2-S |
|  | LIIx R 2-3- | AACGTCTCACAGAGAAGACAACAATGCGGTGAGACCGTCGACCTGCAGACTGGC | LIIc F1-2 RNAi (ccdB) |  |
| LII F 3-4 | LIIx F 3-4+ | TTCGTCTCTTACGGAAGACAAATTGGCGGTGAGACCAAAGCTGAACGAGAAACGTAAAA | LIIc F1-2 RNAi (ccdB) | Esp3I cut-ligation into Xpre-S / Xpre2-S |
|  | LIIx F 3-4- | AACGTCTCACAGAGAAGACAAGACAGACATGAGACCGTCGACCTGCAGACTGGC | LIIc F1-2 RNAi (ccdB) |  |
| LII R 3-4 | LIIx R 3-4+ | TTCGTCTCTTACGGAAGACAAATTGGACATGAGACCAAAGCTGAACGAGAAACGTAAAA | LIIc F1-2 RNAi (ccdB) | Esp3I cut-ligation into Xpre-S / Xpre2-S |
|  | LIIx R 3-4- | AACGTCTCACAGAGAAGACAAGACAGCGGTGAGACCGTCGACCTGCAGACTGGC | LIIc F1-2 RNAi (ccdB) |  |
| LII F 4-5 | LIIx F 4-5+ | TTCGTCTCTTACGGAAGACAATGTCGCGGTGAGACCAAAGCTGAACGAGAAACGTAAAA | LIIc F1-2 RNAi (ccdB) | Esp3I cut-ligation into Xpre-S / Xpre2-S |
|  | LIIx F 4-5- | AACGTCTCACAGAGAAGACAACTCAGACATGAGACCGTCGACCTGCAGACTGGC | LIIc F1-2 RNAi (ccdB) |  |
| LII R 4-5 | LIIx R 4-5+ | TTCGTCTCTTACGGAAGACAATGTCGACATGAGACCAAAGCTGAACGAGAAACGTAAAA | LIIc F1-2 RNAi (ccdB) | Esp3I cut-ligation into Xpre-S / Xpre2-S |
|  | LIIx R 4-5- | AACGTCTCACAGAGAAGACAACTCAGCGGTGAGACCGTCGACCTGCAGACTGGC | LIIc F1-2 RNAi (ccdB) |  |
| LII F 5-6 | LIIx F 5-6+ | TTCGTCTCTTACGGAAGACAATGAGGCGGTGAGACCAAAGCTGAACGAGAAACGTAAAA | LIIc F1-2 RNAi (ccdB) | Esp3I cut-ligation into Xpre-S / Xpre2-S |
|  | LIIx F 5-6- | AACGTCTCACAGAGAAGACAACAGAGACATGAGACCGTCGACCTGCAGACTGGC | LIIc F1-2 RNAi (ccdB) |  |
| LII R 5-6 | LIIx R 5-6+ | TTCGTCTCTTACGGAAGACAATGAGGACATGAGACCAAAGCTGAACGAGAAACGTAAAA | LIIc F1-2 RNAi (ccdB) | Esp3I cut-ligation into Xpre-S / Xpre2-S |
|  | LIIx R 5-6- | AACGTCTCACAGAGAAGACAACAGAGCGGTGAGACCGTCGACCTGCAGACTGGC | LIIc F1-2 RNAi (ccdB) |  |
| LII dy 2-3 | LII 2-3 dy + | GAAGACAAGGGCTAGATAATTAGGCTAAACTATCCTTAAGGATTGTTGTCTTCCCTCGAATTCCCAAGCTTATC | pAMP (Genart®) | amplified with LII dy- |
| LII dy 4-5 | LII 4-5 dy + | GAAGACAATGTCTAGATAATTAGGCTAAACTATCCTTAAGGTGAGTTGTCTTCCCTCGAATTCCCAAGCTTATC | pAMP (Genart®) | amplified with LII dy- |
| LII dy 4-6 | LII 4-6 dy + | GAAGACAATGTCTAGATAATTAGGCTAAACTATCCTTAAGGTCTGTTGTCTTCCCTCGAATTCCCAAGCTTATC | pAMP (Genart®) | amplified with LII dy- |
| LII dy 2-6 | LII 2-6 dy + | TTTGAAGACAAGGGCTAGATAATTAGGCTAAACTATCCTTAAGGTCTGTTGTCTTCCCTCGAATTCCCAAGCTTATC | pAMP (Genart®) | amplified with LII dy- |
| LII dy 1-2 | LII 1-2 dy + | TTTGAAGACAATACGTAGATAATTAGGCTAAACTATCCTTAAGGGGGCTTGTCTTCCCTCGAATTCCCAAGCTTATC | pAMP (Genart®) | amplified with LII dy- |
| LII dy 3-4 | LII 3-4 dy + | TTTGAAGACAAATTGTAGATAATTAGGCTAAACTATCCTTAAGGTGTCTTGTCTTCCCTCGAATTCCCAAGCTTATC | pAMP (Genart®) | amplified with LII dy- |
| LII dy 5-6 | LII 5-6 dy + | TTTGAAGACAATGAGTAGATAATTAGGCTAAACTATCCTTAAGGTCTGTTGTCTTCCCTCGAATTCCCAAGCTTATC | pAMP (Genart®) | amplified with LII dy- |
| LII dy 1-3 | LII 1-3 dy + | TTTGAAGACAATACGTAGATAATTAGGCTAAACTATCCTTAAGGATTGTTGTCTTCCCTCGAATTCCCAAGCTTATC | pAMP (Genart®) | amplified with LII dy- |
|  | LII dy - | AGCTCGAGCGAAGCTTTAGA | pAMP (Genart®) |  |
| LII ins 2-3 | LII ins 2-3 + | GAATATATATATATTCGAATATATATATATTCATTGTTGTCTTCCCTCGAATTC | LII dy 2-3 | amplified with LII ins- |
|  |  |  |  |  |
| LII ins 4-5 | LII ins 4-5 + | GAATATATATATATTCGAATATATATATATTCTGAGTTGTCTTCCCTCGAATT | LII dy 4-5 | amplified with LII ins- |
|  | LII ins - | CCTTAAGGATAGTTTAGCCTAATTATCTA | LII dy 2-3 / LII dy 4-5 |  |
|  |  |  |  |  |
| **LIII vector construction** |  |  |  |  |
| LIII fin | LIII fin + | TTCGTCTCTTACGTTGTCTTCAAAGCTGAACGAGAAACGTAAAA | LIIc F1-2 RNAi (ccdB) | Esp3I cut-ligation into Xpre-k or / Xpre2-k |
|  | LIII fin - | AACGTCTCACAGAAAGTCTTCGTCGACCTGCAGACTGGC | LIIc F1-2 RNAi (ccdB) |  |
| LIII F A-B | LIII X A-B + | TTCGTCTCTTACGGGTCTCAGCGGTACGTTGTCTTCAAAGCTGAACGAGAAACGTAAAA | LIIc F1-2 RNAi (ccdB) | Esp3I cut-ligation into Xpre-k or / Xpre2-k |
|  | LIII X A-B - | AACGTCTCACAGAGGTCTCACAGACAGAAAGTCTTCGTCGACCTGCAGACTGGC | LIIc F1-2 RNAi (ccdB) |  |
| LIII R A-B | LIIIx R A-B+ | TTCGTCTCTTACGGGTCTCAGCGGCAGATTGTCTTCAAAGCTGAACGAGAAACGTAAAA | LIIc F1-2 RNAi (ccdB) | Esp3I cut-ligation into Xpre-k or / Xpre2-k |
|  | LIIIx R A-B- | AACGTCTCACAGAGGTCTCACAGATACGAAGTCTTCGTCGACCTGCAGACTGGC | LIIc F1-2 RNAi (ccdB) |  |
| LIII F C-D | LIIIx F C-D+ | TTCGTCTCTTACGGGTCTCACACCTACGTTGTCTTCAAAGCTGAACGAGAAACGTAAAA | LIIc F1-2 RNAi (ccdB) | Esp3I cut-ligation into Xpre-k or / Xpre2-k |
|  | LIIIx F C-D- | AACGTCTCACAGAGGTCTCACCTTCAGAAAGTCTTCGTCGACCTGCAGACTGGC | LIIc F1-2 RNAi (ccdB) |  |
| LIII R C-D | LIIIx R C-D+ | TTCGTCTCTTACGGGTCTCACACCCAGATTGTCTTCAAAGCTGAACGAGAAACGTAAAA | LIIc F1-2 RNAi (ccdB) | Esp3I cut-ligation into Xpre-k or / Xpre2-k |
|  | LIIIx R C-D- | AACGTCTCACAGAGGTCTCACCTTTACGAAGTCTTCGTCGACCTGCAGACTGGC | LIIc F1-2 RNAi (ccdB) |  |
| LIII F E-F | LIIIx F E-F+ | TTCGTCTCTTACGGGTCTCAAATCTACGTTGTCTTCAAAGCTGAACGAGAAACGTAAAA | LIIc F1-2 RNAi (ccdB) | Esp3I cut-ligation into Xpre-k or / Xpre2-k |
|  | LIIIx F E-F- | AACGTCTCACAGAGGTCTCACTCACAGAAAGTCTTCGTCGACCTGCAGACTGGC | LIIc F1-2 RNAi (ccdB) |  |
| LIII R E-F | LIIIx R E-F+ | TTCGTCTCTTACGGGTCTCAAATCCAGATTGTCTTCAAAGCTGAACGAGAAACGTAAAA | LIIc F1-2 RNAi (ccdB) | Esp3I cut-ligation into Xpre-k or / Xpre2-k |
|  | LIIIx R E-F- | AACGTCTCACAGAGGTCTCACTCATACGAAGTCTTCGTCGACCTGCAGACTGGC | LIIc F1-2 RNAi (ccdB) |  |
|  |  |  |  |  |
|  |  |  |  |  |
|  |  |  |  |  |
| **LI constructs** |  |  |  |  |
| 35S mutagenesis | 35S BsaI + | GGGGTCTCTGCGGAGATTAGCCTTTTCAATTTCAGAAAGAATGC | pGWB2 [[4](#_ENREF_4)] |  |
|  | 35S BsaI - | GGGGTCTCACAGACGTGTTCTCTCCAAATGAAATGAACTTCC | pGWB2 [[4](#_ENREF_4)] |  |
|  | 35S Bsa* 1. + | GGCTTACGCAGCACGTCTCATCAAGACG | pGWB2 [[4](#_ENREF_4)] |  |
|  | 35S Bsa* 1. - | CGTCTTGATGAGACGTGCTGCGTAAGCC | pGWB2 [[4](#_ENREF_4)] |  |
|  | 35S Bpi* 2. + | CAGTCTCAGAAGAGCAAAGGGCAATTGAG | pGWB2 [[4](#_ENREF_4)] |  |
|  | 35S Bpi* 2. - | CTCAATTGCCCTTTGCTCTTCTGAGACTG | pGWB2 [[4](#_ENREF_4)] |  |
|  | 35S Bsa/Bpi* 3. + | CACGACTTCAAAGCAAGTGGATTGATGTG | pGWB2 [[4](#_ENREF_4)] |  |
|  | 35S Bsa*/Bpi* 3. - | GTTGGAACGTGTTCTTTTTCCACGATGC | pGWB2 [[4](#_ENREF_4)] |  |
|  | 35S Esp3I Mut + | CTTACGCAGCACGACTCATCAAGACGATC | pGWB2 [[4](#_ENREF_4)] |  |
|  | 35S Esp3I Mut - | GATCGTCTTGATGAGTCGTGCTGCGTAAG | pGWB2 [[4](#_ENREF_4)] |  |
| LI A-B p35S | A-B 35S + | ATGGTCTCTGCGGAGATTAGCC | 35S mutagenesis | ligation into pUC57 (Gent) |
|  | A-B 35S - | TAGGTCTCACAGACGTGTTCTCTCCAAATGAAATG | 35S mutagenesis |  |
| LI A-C p35S | A-B 35S + | ATGGTCTCTGCGGAGATTAGCC | 35S mutagenesis | ligation into pUC57 (Gent) |
|  | A-C 35S - | TAGGTCTCAGGTGCGTGTTCTCTCCAAATGAAATG | 35S mutagenesis |  |
|  |  |  |  |  |
| LI A-B pUbi | A-B Ubi+ | GGGTCTCTGCGGGGAGAGAGGATTTTGAGGAAA | pUB-GW-HYG [[2](#_ENREF_2)] | ligation into pUC57 (Gent) |
|  | A-B Ubi- | ATGGTCTCACAGACTGTAATCACATCAACAACAGATAAA | pUB-GW-HYG [[2](#_ENREF_2)] |  |
| LI A-B pEF1 | EF1 + | ATGGTCTCAGCGGGTGTATATCGTCCAAGTAAAACCTTC | *A. thaliana* Col-0 genomic DNA | ligation into pUC57 (Gent) |
|  | EF1 - | TAGGTCTCTCAGAGGTTAGAGACTGTCAAACAAATCTG | *A. thaliana* Col-0 genomic DNA |  |
| LI A-B pNOS | pNos + | ATGGTCTCAGCGGGATCATGAGCGGAGAATTAAGG | pBI121 (Clontech) | ligation into pUC57 (Gent) |
|  | pNos - | TAGGTCTCTCAGAAGATCCGGTGCAGATTATTTG | pBI121 (Clontech) |  |
| L0 N-term linker | Link 1 + | ATGAAGACTTTACGGGTCTCAAAGGGAGGTGGAGGAGGTTCTGGAGGCGGTGGAAGT | PCR without template | N-Linker |
|  | Link 1 - | TAGAAGACAAGCTACCTCCGCCACCACTTCCACCGCCTCCAG | PCR without template | N-Linker |
| LI D-E Ceruelan, GFP, YFP, T-Sapphire | C-GFP+ | TAGAAGACAATAGCGTGAGCAAGGGCGAGGAG | p35S_GW_T-Sapph[[5](#_ENREF_5)]; pUB-GW-GFP[[2](#_ENREF_2)],;pAM-PAT-YFP[[6](#_ENREF_6)]; p35S_GW_T-Sapph[[5](#_ENREF_5)] | BpiI cut-ligation with N-linker into LI+BpiI |
|  | C-GFP- | ATGAAGACTTCAGAGGTCTCAGATTTTACTTGTACAGCTCGTCCATG |  |  |
| LI D-E mOrange, mCherry | C-GFP+ | TAGAAGACAATAGCGTGAGCAAGGGCGAGGAG | pC1625_pCAG_mCh_GW [[7](#_ENREF_7)]; p35S_GW_mOrange[[5](#_ENREF_5)] | BpiI cut-ligation with N-linker into LI+BpiI |
|  | mOR 2- | ATGAAGACTTTTTCTTCTGCATTACGGGG |  |  |
|  | mOR 3+ | TAGAAGACAAGAAAACCATGGGCTGGG |  |  |
|  | C-GFP- | ATGAAGACTTCAGAGGTCTCAGATTTTACTTGTACAGCTCGTCCATG |  |  |
| LI C-D Cerulean, GFP, YFP, T-Sapphire , mOrange, mCherry | GFP+ | ATGAAGACTTTACGGGTCTCACACCATGGTGAGCAAGGGCGAG | LI D-E Cerulean, GFP, YFP, T-Sapphire, mOrange, mCherry | BpiI cut-ligation into LI+BpiI |
|  | GFP- nostop | TAGAAGACAACAGAGGTCTCACCTTCTTGTACAGCTCGTCCATGC |  |  |
| L0 C-term linker | Link 2 + | TAGAAGACAACAAGGGTGGAGGAGGTTCTGGAGGCGGTGGAAGT | PCR without template | C-Linker |
|  | Link 2 - | ATGAAGACTTCAGAGGTCTCAGGTGCCGCTACCTCCGCCACCACTTCCACCGCCTCCAG | PCR without template | C-Linker |
| LI B-C Cerulean, GFP, YFP, T-Saphhire, mOrange, mCherry | N-GFP-KOZAK + | ATGAAGACTTTACGGGTCTCA TCTGAACAATGGTGAGCAAGGGCGAG | LI C-D Cerulean; GFP; YFP; T-Sapphire; mOrange; mCherry | BpiI cut-ligation with C-linker into LI+BpiI |
|  | N-GFP - | TAGAAGACAACTTGTACAGCTCGTCCATGC |  |  |
| LI C-D noATG Cerulean, GFP, YFP, T-Saphhire, mOrange, mCherry | GFP+ noATG | ATGAAGACTTTACGGGTCTCACACCGTGAGCAAGGGCGAGGAG | LI C-D Cerulean, GFP, YFP, T-Sapphire, mOrange, mCherry | BpiI cut-ligation into LI+BpiI |
|  | GFP- nostop | TAGAAGACAACAGAGGTCTCACCTTCTTGTACAGCTCGTCCATGC |  |  |
| LI C-D dGFP | desGFP + | ATGGTCTCACACCATGGGCTTAATTAATATAATTAATAATCCA | pLV.CMV.dsEGFP [[8](#_ENREF_8)] | ligation into pUC57 (Gent) |
|  | des GFP- | TAGGTCTCACCTTCACATTGATCCTAGCAGAAGCAC | pLV.CMV.dsEGFP [[8](#_ENREF_8)] |  |
| LI C-D RNAi dGFP | desGFP + | ATGGTCTCACACCATGGGCTTAATTAATATAATTAATAATCCA | pLV.CMV.dsEGFP [[8](#_ENREF_8)] | not subcloned |
|  | Si_desGFP - | TAGGTCTCACCTTGAAGTCGATGCCCTTCAGCT | pLV.CMV.dsEGFP [[8](#_ENREF_8)] |  |
|  |  |  |  |  |
|  |  |  |  |  |
| LI C-D Plastid Prelim. | L0 Pla. 1+ | ATGAAGACTTTACGGGTCTCACACCATGGCAAGCATTGCTGGTTC | *L. japonicus* Gifu genomic DNA | BpiI cut-ligation into LI+BpiI |
|  | L0 Pla. 1- | TTGAAGACTTTTCGATTTCAGGGCTCTCTTTGTTACCTGATGACAACAAGCACCCTTTTGG | *L. japonicus* Gifu genomic DNA |  |
|  | L0 Pla 2+ | TTGAAGACTTCGAAACCCTGATGATTATT | *L. japonicus* Gifu genomic DNA |  |
|  | L0 Pla 2- | ATGAAGACTTCAGAGGTCTCACCTTGGGCCTGAATGGAGCAA | *L. japonicus* Gifu genomic DNA |  |
| LI C-D Plastid-Marker | Pla_mut + | GTTGTCATCAGGTAACAAAGAGAGC | LI C-D Plastid Prelim. | BpiI site removal |
|  | Pla_mut - | AAGCACCCTTTTGGCATG | LI C-D Plastid Prelim. |  |
| LI B-E GUSi | GUSi Koz + | ATGGTCTCATCTGAACAATGGTAGATCTGAGGGTAAA | pIG121-Hm [[9](#_ENREF_9)] | ligation into pUC57 (Gent) |
|  | GUSi Koz - | AAGGTCTCAGATTTCATTGTTTGCC | pIG121-Hm [[9](#_ENREF_9)] |  |
| LI C-D 1-10GFP | GFP1-10 fw | ATGGTCTCACACCATGGTGTCTAAGGGCGAAGA | pUC-sGFPFL (Gene synthesis) | ligation into pUC57 (Gent) |
|  | GFP1-10 rv STOP | TAGGTCTCTCCTTTCACTTTTCGTTAGGGTCCTTGG |  |  |
| LI C-D 1-10GFP noATG | GFP1-10 noATG | ATGGTCTCACACCGTGTCTAAGGGCGAAGAACTC | pUC-sGFPFL (Gene synthesis) | ligation into pUC57 (Gent) |
|  | GFP1-10 rv STOP | TAGGTCTCTCCTTTCACTTTTCGTTAGGGTCCTTGG |  |  |
| LI D-E 1-10GFP | C-sGFP+ | TAGAAGACAATAGCGTGTCTAAGGGCGAAGAACTC | pUC-sGFPFL (Gene synthesis) | ligation into pUC57 (Gent) |
|  | C-sGFP- | ATGAAGACTTCAGAGGTCTCAGATTTTACTTTTCGTTAGGGTCCTTGG |  |  |
| LI C-D 11GFP | GFP11 fw | ATGGTCTCACACCATGGACTACAAGGACGACGATGACA | pUC-sGFPFL (Gene synthesis) | ligation into pUC57 (Gent) |
|  | GFP11 rv | TAGGTCTCTCCTTTTATGTGATTCCGGCGG |  |  |
| LI D-E 11GFP | C-GFP11 fw | ATGGTCTCAAAGGGAGACTACAAGGACGACGATGACA | pUC-sGFPFL (Gene synthesis) | ligation into pUC57 (Gent) |
|  | C-GFP11 rv | TAGGTCTCTGATTTTATGTGATTCCGGCGG |  |  |
| LI B-C N-NLS | N-NLS KOZ + | ATGGTCTCATCTGAACAATGCTGCAGCCTAAGAAGAAG | NLS-YC3.6 [[10](#_ENREF_10)] | ligation into pUC57 (Gent) |
|  | N-NLS rv | TAGGTCTCAGGTGGCGGCCGC | NLS-YC3.6 [[10](#_ENREF_10)] |  |
| LI B-C N-NES (I) | N-NES KOZ b+ | ATGGTCTCATCTGAACAATGCTGCAGAACGAGCTTG | NES-YC3.6 [[10](#_ENREF_10)] | ligation into pUC57 (Gent) |
|  | N-NES b rv | TAGGTCTCAGGTGGCGGCCGC | NES-YC3.6 [[10](#_ENREF_10)] |  |
| LI B-C N-NES (II) | N-NES KOZ a + | ATGGTCTCATCTGAACAATGGCAAATTGTTGCTCTCA | p35S_CPK17G2A_NES [[11](#_ENREF_11)] | ligation into pUC57 (Gent) |
|  | N-NES a rv | TAGGTCTCTGGTGCCTCCTCCAGTCTTGTTAATATCAAGTC | p35S_CPK17G2A_NES [[11](#_ENREF_11)] |  |
| LI D-E C-NLS | C-NLS fw | ATGGTCTCAAAGGGACTGCAGCCTAAGAAGAAGAGAAA | NLS-YC3.6 [[10](#_ENREF_10)] | ligation into pUC57 (Gent) |
|  | C-NLS rv | TAGGTCTCTGATTTTACGCACTCGAGTCGACTCC | NLS-YC3.6 [[10](#_ENREF_10)] |  |
| LI D-E C-NES | C-NES fw | ATGGTCTCAAAGGGACTGCAGAACGAGCTTGCTCT | NES-YC3.6 [[10](#_ENREF_10)] | ligation into pUC57 (Gent) |
|  | C-NES rv | TAGGTCTCTGATTTTACGCACTCGAGTCGACTCC | NES-YC3.6 [[10](#_ENREF_10)] |  |
| LI E-F HSP-T | HSP-T fw | ATGGTCTCAAATCATATGAAGATGAAGATGAAA | *A. thaliana* Col-0 genomic DNA | ligation into pUC57 (Gent) |
|  | HSP-T rv | TAGGTCTCTCTCACTTATCTTTAATCATATTCC | *A. thaliana* Col-0 genomic DNA |  |
| LI E-F nos-T | Nos-Term BsaI + | GGGGTCTCTAATCGATCGTTCAAACATTTGGCAATAA | pBI121 (Clontech) | ligation into pUC57 (Gent) |
|  | Nos-Term BsaI - | GGGGTCTCACTCAGATCTAGTAACATAGATGACAC | pBI121 (Clontech) |  |
| LI E-F 35S-T | 35S terminator + | ATGAAGACTTTACGGGTCTCAAATCCGGCCATGCTAGAGTCCG | pK7RWG2 [[3](#_ENREF_3)] | ligation into pUC57 (Gent) |
|  | 35S terminator - | ATGAAGACTTCAGAGGTCTCACTCAAGGTCACTGGATTTTGGTTTT | pK7RWG2 [[3](#_ENREF_3)] |  |
| LI F-G Hygro / Prelim | hyg F1 + | ATGAAGACTTTACGGGTCTCATGAGAGATTAGCCTTTTCAATTTCAGAAA | pUB-GW-HYG [[2](#_ENREF_2)] | BpiI cut-ligation into LI+BpiI |
|  | hyg F1 - | TAGAAGACAAGGTGCGTGTTCTCTCCAAATGAAATG | pUB-GW-HYG [[2](#_ENREF_2)] |  |
|  | hyg F2+ | TAGAAGACAACACCATGAAAAAGCCTGAACTCACC | pUB-GW-HYG [[2](#_ENREF_2)] |  |
|  | hyg F2- | ATGAAGACTTCAGAGGTCTCAGACATGCGGACGTTTTTAATGTACTG | pUB-GW-HYG [[2](#_ENREF_2)] |  |
| LI F-G Hygro | G94 mut + | GTCCGACCTGATGCAGCTCT | LI F-G Hygro / Prelim | removal of Esp3I site |
|  | G94 mut- | ACGCTGTCGAACTTTTCGAT | LI F-G Hygro / Prelim |  |
| LI F-G neo | Neo + | GGTCTCTTGAGGATCATGAGCGGAGAATTAAG | pBI121 (Clontech) | ligation into pUC57 (Gent) |
|  | Neo - | GGTCTCAGACACCCGATCTAGTAACATAGATG | pBI121 (Clontech) |  |
| LI B-C 6xHIS | N-Term 6xHis + | ATGGTCTCATCTGaacaATGCATCACCATCACCATCACGGCGGCAGCGGCGGATCCAGCACCAGAGACCGGGCCCGTCGACTGC | pUC57 (Gent) | amplified with LI dy- |
| LI D-E 6xHIS | C-Term 6xHis + | ATGGTCTCAAAGGGCGGCAGCGGCGGATCCCATCACCATCACCATCACTAAAATCAGAGACCGGGCCCGTCGACTGC | pUC57 (Gent) | amplified with LI dy- |
| LI B-C HA | N-Term HA + | ATGGTCTCATCTGaacaATGTACCCATACGATGTTCCAGATTACGCTGGCGGCAGCGGCGGATCCAGCACCAGAGACCGGGCCCGTCGACTGC | pUC57 (Gent) | amplified with LI dy- |
| LI D-E HA | C-Term HA + | ATGGTCTCAAAGGGCGGCAGCGGCGGATCCTACCCATACGATGTTCCAGATTACGCTTAAAATCAGAGACCGGGCCCGTCGACTGC | pUC57 (Gent) | amplified with LI dy- |
| LI B-C c-myc | N-Term c-myc + | ATGGTCTCATCTGaacaATGGAGCAAAAGTTGATTTCTGAGGAGGATCTTGGCGGCAGCGGCGGATCCAGCACCAGAGACCGGGCCCGTCGACTGC | pUC57 (Gent) | amplified with LI dy- |
| LI D-E c-myc | C-Term c-myc + | ATGGTCTCAAAGGGCGGCAGCGGCGGATCCGAGCAAAAGTTGATTTCTGAGGAGGATCTTTAAAATCAGAGACCGGGCCCGTCGACTGC | pUC57 (Gent) | amplified with LI dy- |
|  | LI dy - | GGGATCCGATATCTAGATGC | pUC57 (Gent) |  |
|  |  |  |  |  |
| **Custom backbone vector dummies** | |  |  |  |
| LI dy Esp3I-lacZ A-B | lacZ-Pro fw | TTGGTCTCTGCGGTGAGACGTACCGCCTTTGAGTGAGCTG | pUC57 (Genscript) | ligation into pUC57 (Gent) |
|  | lacZ-Pro rv | AAGGTCTCACAGAAGAGACGATGCCGGGAGCAGACA | pUC57 (Genscript) |  |
| LI dy Esp3I-lacZ C-D | lacZ-GOI fw | TTGGTCTCTCACCTGAGACGTACCGCCTTTGAGTGAGCTG | pUC57 (Genscript) | ligation into pUC57 (Gent) |
|  | lacZ GOI rv | AAGGTCTCACCTTAGAGACGATGCCGGGAGCAGACA | pUC57 (Genscript) |  |
| LI dy Esp3I-ccdB A-B | ccdB - Pro fw | TTCGTCTCTGCGGTGAGACCAAAGCTGAACGAGAAACGTAAAA | LIIc F 1-2 RNAi | ligation into pUC57 (Gent) |
|  | ccdB - Pro rv | AACGTCTCACAGAAGAGACCGTCGACCTGCAGACTGGC | LIIc F 1-2 RNAi |  |
| LI dy Esp3I-ccdB C-D | ccdb GOI fw | TTCGTCTCTCACCTGAGACCAAAGCTGAACGAGAAACGTAAAA | LIIc F 1-2 RNAi | ligation into pUC57 (Gent) |
|  | ccdB GOI rv | AACGTCTCACCTTAGAGACCGTCGACCTGCAGACTGGC | LIIc F 1-2 RNAi |  |
|  |  |  |  |  |
| **Silencing Vectors** |  |  |  |  |
| LI Intron | Intron fw | ATGGTCTCAAAGGAATTCCTGTGGTTGGAGAAGC | pUB-GWS-GFP [[2](#_ENREF_2)] |  |
|  | Intron rv | TAGGTCTCTAAGGAGCTTCTCCTCCTCTGCTAACG | pUB-GWS-GFP [[2](#_ENREF_2)] |  |
| LI B-E Esp3I dy | LI Esp3I dy fw | ATGGTCTCATCTGTGAGACGTTCGTCTCAAATCAGAGACCTGCAAGCTTGGCGTAATCAT | pUC57 (Genscript) | inserted into LII RNAi prelimiary vector |
|  | LI dy - | P-GGGATCCGATATCTAGATGC | pUC57 (Genscript) | inserted into LII RNAi prelimiary vector |
| Esp3I - ccdB C-C dy 1 of 2 | ccdb 1 fw | ATCGTCTCATCTGCACCTGAGACCAAAGCTGAACGAGAAACGTAAAA | pENTR-BsaI | inserted into LII RNAi final vector |
|  | ccdb 2 rv | TACGTCTCTACACGAAAAACATATTCTCAATAAACC | pENTR-BsaI |  |
| Esp3I - ccdB C-C dy 2 of 2 | ccdb 2 fw | ATCGTCTCAGTGTCAGCCAATCCCTGG | pENTR-BsaI |  |
|  | ccdb 3 rv | TACGTCTCTGATTCACCTGAGACCGTCGACCTGCAGACTGGC | pENTR-BsaI |  |

**References**

1. Kovach ME, Elzer PH, Steven Hill D, Robertson GT, Farris MA, et al. (1995) Four new derivatives of the broad-host-range cloning vector pBBR1MCS, carrying different antibiotic-resistance cassettes. Gene 166: 175-176.

2. Maekawa T, Kusakabe M, Shimoda Y, Sato S, Tabata S, et al. (2008) Polyubiquitin promoter-based binary vectors for overexpression and gene silencing in *Lotus japonicus*. Molecular Plant-Microbe Interactions 21: 375-382.

3. Karimi M, Inzé D, Depicker A (2002) GATEWAY vectors for *Agrobacterium*-mediated plant transformation. Trends in plant science 7: 193-195.

4. Nakagawa T, Kurose T, Hino T, Tanaka K, Kawamukai M, et al. (2007) Development of series of gateway binary vectors, pGWBs, for realizing efficient construction of fusion genes for plant transformation. Journal of Bioscience and Bioengineering 104: 34-41.

5. Bayle V, Nussaume L, Bhat RA (2008) Combination of novel green fluorescent protein mutant TSapphire and DsRed variant mOrange to set up a versatile in planta FRET-FLIM assay. Plant Physiology 148: 51-60.

6. Lefebvre B, Timmers T, Mbengue M, Moreau S, Hervé C, et al. (2010) A remorin protein interacts with symbiotic receptors and regulates bacterial infection. Proceedings of the National Academy of Sciences 107: 2343-2348.

7. Bultmann S, Morbitzer R, Schmidt CS, Thanisch K, Spada F, et al. (2012) Targeted transcriptional activation of silent oct4 pluripotency gene by combining designer TALEs and inhibition of epigenetic modifiers. Nucleic Acids Research.

8. Mussolino C, Morbitzer R, Lütge F, Dannemann N, Lahaye T, et al. (2011) A novel TALE nuclease scaffold enables high genome editing activity in combination with low toxicity. Nucleic Acids Research 39: 9283-9293.

9. Hiei Y, Ohta S, Komari T, Kumashiro T (1994) Efficient transformation of rice (*Oryza sativa L*.) mediated by *Agrobacterium* and sequence analysis of the boundaries of the T-DNA. Plant Journal 6: 271-282.

10. Krebs M, Held K, Binder A, Hashimoto K, Den Herder G, et al. (2012) FRET-based genetically encoded sensors allow high-resolution live cell imaging of Ca2+ dynamics. Plant Journal 69: 181-192.

11. Mehlmer N, Parvin N, Hurst CH, Knight MR, Teige M, et al. (2012) A toolset of aequorin expression vectors for in planta studies of subcellular calcium concentrations in *Arabidopsis thaliana*. Journal of Experimental Botany 63: 1751-1761.
